# Defective replication initiation results in locus specific chromosome breakage and a ribosomal RNA deficiency in yeast

**DOI:** 10.1371/journal.pgen.1007041

**Published:** 2017-10-16

**Authors:** Joseph C. Sanchez, Elizabeth X. Kwan, Thomas J. Pohl, Haley M. Amemiya, M. K. Raghuraman, Bonita J. Brewer

**Affiliations:** 1 Molecular and Cellular Biology Program, University of Washington, Seattle, WA, United States of America; 2 Department of Genome Sciences, University of Washington, Seattle, WA, United States of America; MRC Laboratory of Molecular Biology, UNITED KINGDOM

## Abstract

A form of dwarfism known as Meier-Gorlin syndrome (MGS) is caused by recessive mutations in one of six different genes (*ORC1*, *ORC4*, *ORC6*, *CDC6*, *CDT1*, and *MCM5*). These genes encode components of the pre-replication complex, which assembles at origins of replication prior to S phase. Also, variants in two additional replication initiation genes have joined the list of causative mutations for MGS (Geminin and *CDC45*). The identity of the causative MGS genetic variants strongly suggests that some aspect of replication is amiss in MGS patients; however, little evidence has been obtained regarding what aspect of chromosome replication is faulty. Since the site of one of the missense mutations in the human *ORC4* alleles is conserved between humans and yeast, we sought to determine in what way this single amino acid change affects the process of chromosome replication, by introducing the comparable mutation into yeast (*orc4*^Y232C^). We find that yeast cells with the *orc4*^Y232C^ allele have a prolonged S-phase, due to compromised replication initiation at the ribosomal DNA (rDNA) locus located on chromosome XII. The inability to initiate replication at the rDNA locus results in chromosome breakage and a severely reduced rDNA copy number in the survivors, presumably helping to ensure complete replication of chromosome XII. Although reducing rDNA copy number may help ensure complete chromosome replication, *orc4*^Y232C^ cells struggle to meet the high demand for ribosomal RNA synthesis. This finding provides additional evidence linking two essential cellular pathways—DNA replication and ribosome biogenesis.

## Author summary

The origin recognition complex (ORC) is essential for licensing replication origins during M/G1 for their firing in the subsequent S phase. Individuals with a rare form of dwarfism called Meier-Gorlin syndrome (MGS) have mutations in proteins required for origin activation, including various subunits of ORC. To better understand the molecular and cellular consequences of these mutations, we introduced an equivalent MGS mutation in *ORC4* into yeast. We find that origin activity in the ribosomal DNA (rDNA) repeats is severely compromised in yeast that harbor the MGS allele. Consequently, cells that have reduced their rDNA copy number from ~150 to fewer than 30 copies overtake the culture. Although the loss of rDNA repeats helps ensure the complete replication of chromosome XII during S phase, cells with fewer rDNA repeats struggle to meet the high demand for ribosomal RNA.

## Introduction

The faithful and timely duplication of a cell’s genome is required every round of division. During eukaryotic S phase, DNA replication initiates at multiple sites along each chromosome called origins of replication. Eukaryotic replication initiation has been best characterized in the budding yeast *Saccharomyces cerevisiae*, where chromosomal origins were first identified by their ability to maintain recombinant plasmids after transformation into yeast [1]. The majority of these Autonomous Replication Sequences or ARS elements correspond to the ~300 chromosomal origins of replication that are scattered across the genome and share a core consensus sequence called the ACS (ARS consensus sequence) [2]. Subsequent biochemical and genetic work in yeast identified many of the essential genes for replication initiation [3–5]. Features that define origins in higher eukaryotes differ significantly from yeast ARSs, but the proteins that carry out origin recognition and initiation are strikingly conserved in sequence and structure across eukaryotes (S1 Fig) [6–9].

In budding yeast, a six-membered protein complex called the Origin Recognition Complex (Orc1-6) binds ARSs throughout the cell cycle [10]. To become competent (or licensed) for initiation, additional proteins are recruited by ORC during the M and G1 phases [11]. The first licensing factor to bind is Cdc6, which facilitates the recruitment of the Mcm2-7 helicase component through an interaction with Cdt1 [9,12–16]. Collectively, this protein complex is known as the Pre-Replication Complex (Pre-RC) and, once assembled on an origin, licenses it to initiate DNA replication or “fire” in the subsequent S phase. During the onset of S phase CDK- and DDK-dependent phosphorylation events complete assembly of the replisomes, including two helicase complexes, allowing replication to proceed bi-directionally from the origin of replication [17–19].

As chromosome replication is essential for cell division, there has been a tacit assumption that mutations that impair the function of proteins involved in DNA replication would be incompatible with metazoan life. Yet, researchers reported in 2011 that amino acid substitutions in proteins involved in the initiation of DNA replication, proteins first identified in *S*. *cerevisiae*, are responsible for a form of proportionate dwarfism called Meier-Gorlin syndrome (MGS) [20,21]. Individuals with MGS have phenotypes that include short stature, small external ears and missing or underdeveloped kneecaps [22,23]—phenotypes not obviously associated with chromosome replication defects.

The specific genetic variants found in patients with MGS include homozygous or compound heterozygous alterations in six different Pre-RC genes (*ORC1*, *ORC4*, *ORC6*, *CDT1*, *CDC6*, and *MCM5*) [20,21,24]. Recent work has identified *de novo* autosomal dominant mutations in Geminin (encoded by *GMNN*), an inhibitor of DNA replication that is unique to higher eukaryotes [25]. Additionally, biallelic mutations in *CDC45*, which is required for both origin initiation and elongation during S phase, have been found to be causative for some cases of MGS [26]. Considering the known roles of these proteins in origin initiation, a reasonable hypothesis is that these mutations are adversely affecting DNA replication and thus reducing cell proliferation so that individuals harboring these variants are uncommonly small. Consistent with this hypothesis, previous work using Epstein-Barr virus replication as an assay has found that immortalized fibroblasts and cultured lymphoblastoid cells derived from MGS patients are diminished in their ability to initiate replication [27,28]; however, defects in replication initiation did not always correlate with slowed S phase in these cells [27]. Additionally, MGS mutations have been shown to affect aspects of cell biology other than DNA replication, such as centrosome duplication and cilia formation [27,29]. The defective cilia formation phenotype observed in MGS cells is thought to contribute to some of the developmental abnormalities associated with this condition [27]. Although the proteins linked with MGS have been studied extensively in yeast and other eukaryotes, it is not clear how MGS mutations might affect chromosome replication to give rise to the phenotypes observed in humans. Therefore, understanding how MGS mutations affect chromosome replication may shed light on how they contribute to the phenotypes in humans.

In this study, we have replaced the genomic copy of the budding yeast *ORC4* with a mutated version (*orc4*^Y232C^) bearing a tyrosine-to-cysteine change that is orthologous to the Tyr174Cys mutation reported in human patients (S2A and S2B Fig) [21]. We find that yeast cells bearing this *orc4*^Y232C^ allele have a longer cell cycle time that is mostly accounted for by a lengthened S phase. Additionally, we find that in *orc4*^Y232C^ cells more than 85% of the earliest firing origins are unaltered in their time and/or efficiency; however, the origins present in each copy of the ribosomal DNA (rDNA) are severely compromised in their ability to fire and the number of copies of the rDNA repeat drops from ~150 to as few as 10. Previous work with *ORC1* and *ORC2* temperature sensitive mutants also revealed shrinkage of the rDNA; however, that work did not provide a model for how the copy number reduction occurred and proposed a checkpoint control of genome-wide replication initiation as an explanation for loss of rDNA repeats [30]. Our findings with the *orc4*^Y232C^ allele reveal that the mechanism for rDNA copy number loss is chromosome XII breakage as a consequence of the “random replication gap” problem and that insufficient replication initiation outside of the rDNA locus is not likely the cause for rDNA shrinkage. Furthermore, we show that the reduction in rDNA copy number, by restricting rRNA synthesis, constrains the translational capacity, possibly explaining the slow growth observed in *orc4*^Y232C^ cells. While it remains to be seen whether these phenotypes are also common to Meier-Gorlin patient cells, our characterization of the *orc4*^Y232C^ allele in *S*. *cerevisiae* highlights an unsuspected pathway linking replication dysfunction and growth control.

## Results

### The slow growth of yeast with the *orc4*^Y232C^ allele is due to a delay in completing S phase

**Fig 1:**
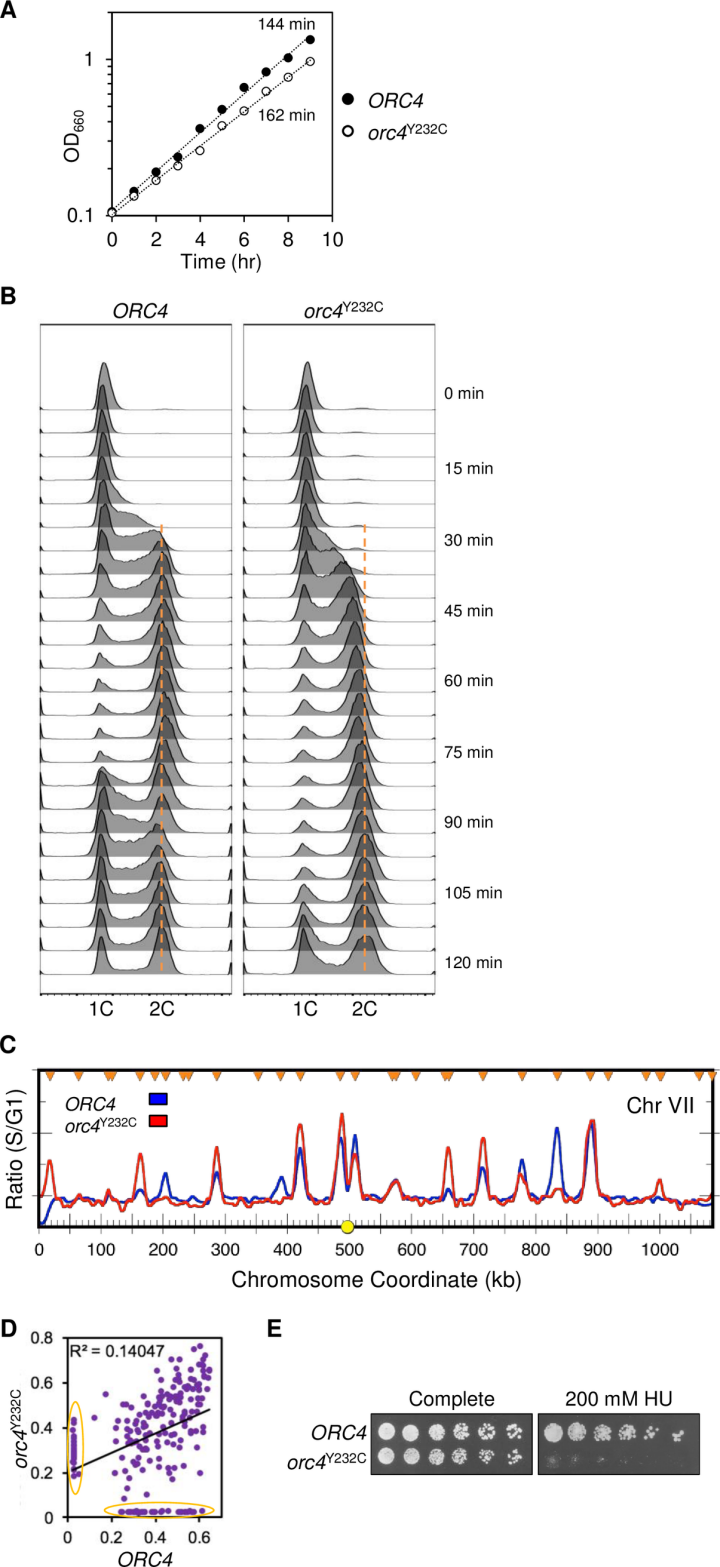
Growth and cell cycle characterization of *orc4*^Y232C^ cells. (A) Growth of *ORC4* and *orc4*^Y232C^ cells as measured by change in optical density (OD_660_) of mid-log phase cultures in synthetic complete medium at 30°C. The mutant (white circle) shows a modest growth defect with a doubling-time 18 minutes (12%) longer than wild-type cells (black circle). (B) S phase progression of *ORC4* (left) and *orc4*^Y232C^ (right) cells as measured by flow cytometry. Cells were synchronously released into S phase and cell samples were collected at 5-minute intervals. *ORC4* cells enter S phase ~20 minutes after release from alpha-factor, whereas *orc4*^Y232C^ cells entered S phase ~25 minutes after release. By 85 minutes *ORC4* cells are cycling back to begin a new cell cycle, while *orc4*^Y232C^ cells do not begin to cycle back until 120 minutes. The orange dotted line indicates the expected DNA content for cells that have completed replication. (C) ssDNA profiles of early origin activity in *ORC4* (blue) compared to *orc4*^Y232C^ (red) cells are shown for Chr VII. G1 cells were synchronously released into S phase in the presence of HU to reduce movement of replisomes away from origins of replication. The y-axis values represent the relative amounts of ssDNA calculated as the ratio of fluorescent signal of S phase sample (30 min) to G1 control. Chromosome coordinates for Chr VII are displayed along the x-axis and sites of origin initiation appear as peaks along this axis. The centromere location is represented as a yellow circle on the x-axis and verified origins of replication are marked by orange triangles. See S3 Fig for the full set of profiles. (D) Scatter plot comparing the areas under the peaks of ssDNA observed in *ORC4* vs. *orc4*^Y232C^ cells. Origins whose activity was largely restricted to one genotype or the other are encircled by the orange ovals. (E) Sensitivity of *orc4*^Y232C^ cells to hydroxyurea (HU). Serial dilutions (1:3) of cells were plated on synthetic complete medium with or without HU (200 mM) and incubated at 30°C for 3 days.

A missense mutation in *ORC4* has been shown to be causative for some instances of MGS in humans [21]. This specific mutation results in an amino acid substitution (Tyrosine to Cysteine) at position 174 of the human Orc4 protein and occurs in a region with homology to the AAA+ (ATPases Associated with diverse cellular Activities) related domain of the *S*. *cerevisiae* Orc4p [21]. The initial work investigating the equivalent MGS mutation (*orc4*^Y232C^) in *S*. *cerevisiae* revealed a slow-growth phenotype [21]. In that experiment, the investigators constructed a strain that had the *orc4*^Y232C^ allele on a plasmid rescuing the inviable chromosomal deletion of *ORC4*. Because the *orc4*^Y232C^ allele was not in its native location, it was not clear to what extent the slow growth was due to plasmid loss or poor expression from the plasmid versus other, more wide-spread defects in chromosome replication or segregation. To explore in more detail the consequences of the *orc4*^Y232C^ mutation, we replaced the wild type yeast *ORC4* allele with the MGS equivalent allele *orc4*^Y232C^ at its native chromosomal locus. Cells with the chromosomal *orc4*^Y232C^ allele grow more slowly than wild type (population doubling time of 2.7 hr. vs. 2.4 hr; Fig 1A). The more marked difference in growth rates reported for the strains analyzed by Guernsey *et al*. [21] is probably due to loss of the plasmid bearing the sole source of Orc4p in their experiments.

To explore the slow growth phenotype, we performed flow cytometry on cells synchronously proceeding through the cell cycle after having been arrested at START by treatment with alpha-factor (Fig 1B). Initiation of S phase appears to be slightly delayed in the mutant but after replicating much of its genomic DNA the mutant shows a more pronounced delay in reaching a full G2 DNA content and progressing back into G1. We reasoned that a delay in S phase entry could result if the *orc4*^Y232C^ mutation altered early origin firing. To examine this possibility we used an assay that specifically examines early origin activation across the genome [31]. This assay uses microarray hybridization to measure the levels of single stranded DNA exposed at replication forks. Both the peak position and peak amplitude of ssDNA formed at genomic loci are informative. While we do not fully understand the molecular processes that give rise to peaks of different amplitudes—e.g., number of cells that have activated a particular origin vs. amount of ssDNA revealed at different forks—the results from different replicates of the experiment are highly reproducible. We find that origins that are known to fire early and are efficient produce the peaks of greatest magnitude, while later firing and less efficient origins produce smaller or no peaks in this assay [31].

We carried out the ssDNA assay on the wild type *ORC4* and *orc4*^Y232C^ mutant and a representative comparison of the two is shown in Fig 1C (to view comparisons of all chromosomes see S3 Fig). We observed differences in origin usage in *ORC4* compared to *orc4*^Y232C^ and could discern three classes of origins. Some origins had comparable ssDNA peaks in both strains (cf. origins at 420 kb, 490 kb, 890 kb). Some origins that were active in *ORC4* showed significant reduction in ssDNA accumulation in *orc4*^Y232C^ (cf. 200 kb, 390 kb, 830 kb). Perhaps most surprisingly, the converse was also true: some origins that are late or inefficiently initiated in *ORC4* cells had significant ssDNA peaks in the *orc4*^Y232C^ mutant, indicating earlier or more efficient initiation than in wild type (cf. 15 kb, 170 kb, 670 kb). To assess quantitatively the magnitude in change to the early replication program of origin firing, we quantified the area under the peaks observed in the ssDNA assays for *ORC4* and *orc4*^Y232^. Peak areas in *ORC4* vs. *orc4*^Y232C^ were highly discordant (Fig 1D; R^2^ = ~0.14). These results highlight substantial differences in the early origin firing of origins in *orc4*^Y232^ cells: of the 213 origins represented in the scatter plot (Fig 1D), 31 are significantly depressed or delayed in activity in the *orc4*^Y232C^ mutant (x-axis: outlined in orange) while another 24 origins are now detected as firing early (y-axis: outlined in orange). However, overall, the number of early firing origins was similar between wild type and *orc4*^Y232^ cells. While the ssDNA assay limits us to monitoring only early-firing origins we anticipate that even if no more origin activation occurred (as is the case for cells with a deletion of *CLB5*, the major S phase cyclin), the completion of S phase would only be delayed by an additional 15 minutes [32].

The flow cytometry data of a synchronous culture of *orc4*^Y232C^ cells showed more than a 15 minute delay in completion of S phase and cell division (Fig 1B). For example, by 85 minutes wild type cells had divided and were undergoing a second round of DNA synthesis. The mutant cells do not show a comparable DNA content profile until 120 minutes after release. We hypothesized that this 35 minute delay could be due to cells encountering problems in late S phase and that the gradual shift to 2C DNA content results from slow progress in completing genomic DNA replication. Interestingly, patient-derived lymphoblastoid cell lines harboring an *ORC4*-MGS mutation also exhibit a delayed S phase transit [27]. An alternative hypothesis for the gradual shift to 2C DNA content that is observed in *orc4*^*Y252C*^ cells may be that they have completed genome replication on time and are continuing to replicate mitochondrial DNA but are delaying the G2/M transition for other reasons. However, the observation that cells with the *orc4*^*Y252C*^ mutation are unable to form colonies on plates with 200 mM hydroxyurea (HU; Fig 1E), a drug that inhibits ribonucleotide reductase and thereby slows the progress of replication forks, supports the hypothesis that the cell cycle delay stems from a chromosome replication defect.

### The *orc4*^Y232C^ mutation results in reduced copy number and origin activity at the rDNA locus

**Fig 2:**
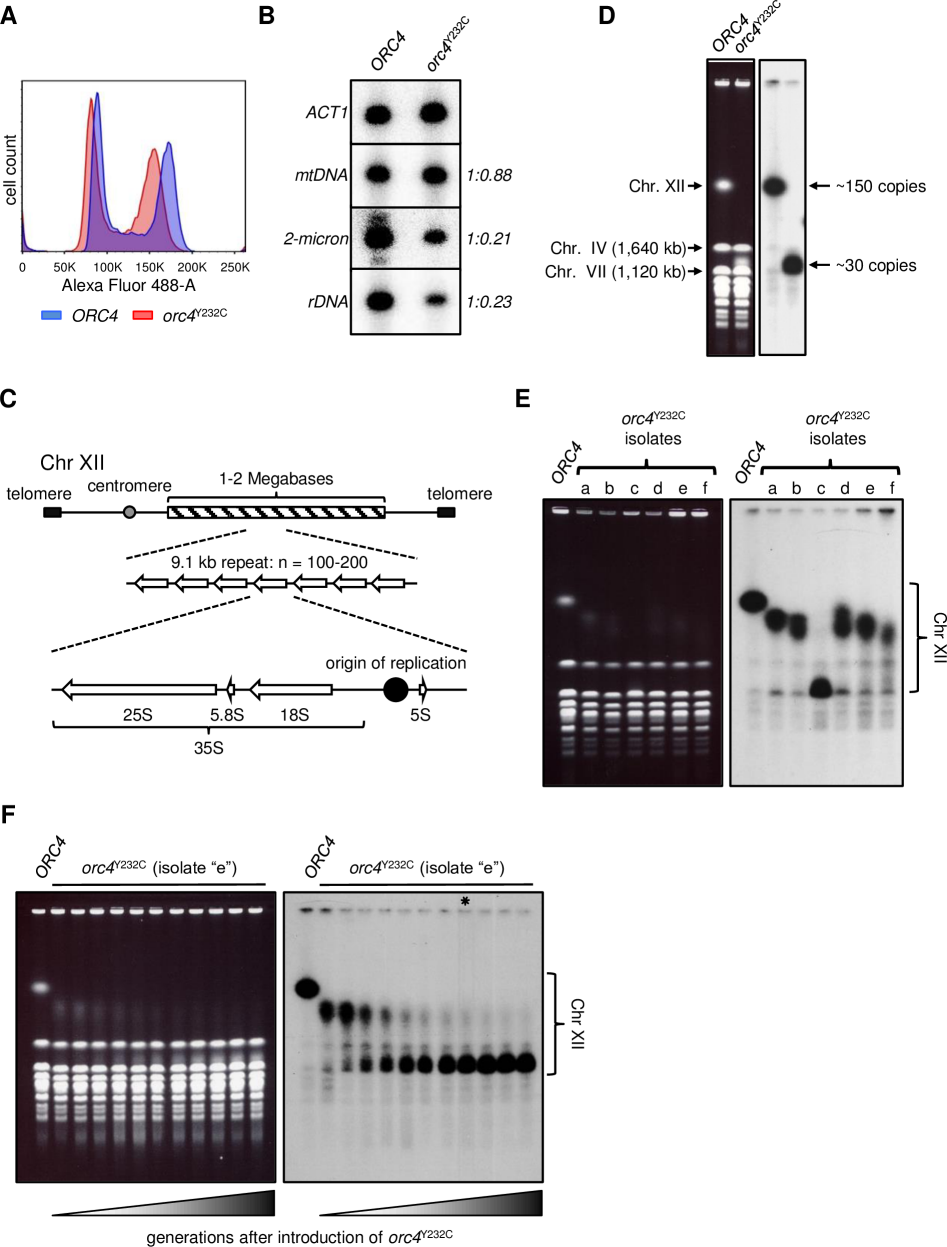
Reduction of rDNA copy number in *orc4*^Y232C^ cells. (A) Flow cytometry histograms of log-phase populations of *ORC4* (blue) and *orc4*^Y232C^ (red) cells overlaid with one another. The mutant’s profile is shifted to the left indicating a loss (~10%) of cellular DNA content. (B) Quantitative Southern blot analysis of repetitive DNA sequences. Genomic DNA from *ORC4* and *orc4*^Y232C^ cells was digested with a restriction enzyme and separated by standard gel electrophoresis. The Southern blot of the gel was first probed with *ACT1* sequence (as a single copy control) and then subsequently probed with various repetitive DNA sequence including mitochondrial DNA, the native, nuclear 2-micron plasmid DNA, and rDNA. The bands above and below the major center band present when probed for 2-micron correspond to restriction fragments from the low levels of naturally occurring dimeric plasmid molecules between the A and B isomers of the plasmid. (C) Cartoon depiction of the yeast rDNA locus. The yeast rDNA locus consists of 100–200 copies of a 9.1 kb tandem repeat located on Chr XII. Each rDNA repeat encodes the template necessary to make ribosomal RNA (25S, 5.8S, 18S and 5S) and also contains an origin of replication. (D) CHEF gel analysis of Chr XII size in *ORC4* and *orc4*^Y232C^ cells. Left panel, ethidium bromide stained image; right panel, image of Southern blot following hybridization with a Chr XII-specific single-copy sequence. The faster migration of Chr XII in *orc4*^Y232C^ cells confirms the loss of chromosomal rDNA repeats from ~150 (*ORC4*) to ~30 copies (*orc4*^Y232C^). (E) CHEF gel analysis of variation in rDNA copy number in six additional isolates (a-f) of *orc4*^Y232C^. Left, ethidium bromide stained image; right, Southern blot hybridization for Chr XII as in (D). Samples were prepared after ~20 generations of growth following introduction of the *orc4*^Y232C^ mutation. All six isolates had a smaller Chr XII than *ORC4* due to loss of rDNA repeats. The rDNA copy number in the isolates ranges from ~30 copies (isolate c) to ~100 copies (isolates a, b, d, and e). (F) Long-term growth of isolate “e” shows rDNA copy number stabilizes at ~30 copies. After confirming isolate “e” to have the mutant *orc4*^Y232C^ allele, cells were continuously passaged for ~100 generations in batch culture. Cells were allowed to grow to saturation after each passage. For each sample cells were collected and whole chromosomes were separated by CHEF gel electrophoresis (ethidium bromide stained image on left, Southern hybridization for Chr XII). By ~80 generations (*) the rDNA copy number stabilized at ~30 copies in the population.

When comparing the flow cytometry profiles of the *orc4*^Y232C^ cells relative to their wild type *ORC4* control cells, we noticed a consistent shift to an approximately 10% lower DNA content for the *orc4*^Y232C^ cells (Fig 2A) regardless of their cell cycle phase. Considering that a haploid yeast genome is ~13 Mb in size, this difference in peak location means that ~1.3 Mb of sequence is missing from the mutant. Since the *ORC4* and *orc4*^Y232C^ strains analyzed in this experiment are haploid, the difference in DNA content cannot be ascribed to chromosome aneuploidy. As an alternative possibility, we investigated whether loss of repetitive sequences could explain the shift in the mutant’s flow cytometry histogram. Candidate repetitive DNA species included mitochondrial DNA (mtDNA; ~85 kb, ~50 copies per cell), the native 2-micron plasmid (6.3 kb, ~50 copies per cells), and ribosomal DNA (rDNA; 9.1 kb, 100–200 copies per cell) [33–35]. The 2-micron plasmid and mitochondrial genomes are autonomous elements and copy number variation can occur through failure of replication and/or segregation [33,36]. In contrast, the rDNA locus is a tandemly repeated array located on chromosome XII and copy number changes occur through homologous inter- or intra-chromosome recombination [37,38]. To determine which, if any, of these repeated sequences might account for the missing genomic DNA content of the *orc4*^Y232C^ strain we performed quantitative Southern blotting of the mutant and wild type strains. We found that the mutant cells retained most (~88%) of their mtDNA (whose replication is ORC-independent), but retained only ~21% of the 2-micron plasmid (a selfish DNA element that is dependent on ORC for maintenance [39]) and ~23% of their rDNA copies relative to the wild type cells (Fig 2B). The small decrease in mtDNA cannot account for the shift observed in the flow cytometry profiles; however, together the loss of 2-micron and rDNA repeats is sufficient to account for the missing DNA content. Reduction of the 2-micron plasmid would have no significant impact on the cell’s health as cells completely cured of their 2-micron plasmids actually enjoy a slight selective growth advantage [40]. However, the reduction in the rDNA locus could have a more significant impact on cell physiology.

The rDNA locus comprises over half of the physical length of chromosome XII and accounts for ~10% of the nuclear genome (Fig 2C) [41]. Each 9.1 kb repeat contains the template for making ribosomal RNAs (rRNAs), the main structural component of ribosomes [42]. Based on the quantitative Southern blot analysis, we estimate that the rDNA copy number was reduced from ~150 copies in wild type cells to ~30 copies in the mutant. CHEF gel analysis of whole yeast chromosomes confirmed the expected size reduction of chromosome XII in the mutant (Fig 2D). Thus, the deficit of ~120 copies of rDNA in the mutant would account for ~1.1 Mb of the missing sequence, with the ~40 fewer copies of 2-micron plasmid (~252 kb) accounting for the remainder of the missing ~1.3 Mb.

Since the shrinkage of chromosome XII in *orc4*^Y232C^ cells was such a striking phenomenon, we tested six additional isolates of the mutant to determine if this size reduction was a consistent phenotype of the *orc4*^Y232C^ mutation. Immediately after selection for loss of the *ORC4* allele (~20 generations), all six isolates had a smaller chromosome XII than wild type, with an rDNA locus of ~30–100 repeats (Fig 2E). To determine the stability of the rDNA locus in the different *orc4*^Y232C^ isolates over time we monitored the size of their chromosome XII after long-term growth of the strains. We found that after ~100 generations, all six populations had arrived at the same rDNA copy number of ~30 repeats (S4 Fig), suggesting that the *orc4*^Y232C^ mutation imposes strong selection for cells that have reduced their rDNA copy number. To determine the dynamics of rDNA repeat loss we analyzed samples collected during the ~100 generations of growth for one isolate (e) (Fig 2F). Rather than a steady decrease in rDNA copy number during propagation of the culture, we observed a decreasing abundance of the original chromosome XII size with concomitant increased abundance of the final size, with no obvious populations with intermediate sizes. These results support the hypothesis that a sub-population of cells with the final rDNA copy number already existed in the initial culture or appeared shortly thereafter, and had a selective advantage over cells with the longer rDNA. The descendants of the decreased-rDNA variants eventually took over the population, approaching fixation by ~80 generations of growth (Fig 2F; indicated by the lane marked with an asterisk).

Next, we asked if the selection for a loss of rDNA repeats was a phenotype specific to the *orc4*^Y232C^ allele. To test this hypothesis, we introduced a different MGS-like mutation into budding yeast. Some cases of MGS have been reported to be caused by mutations in *CDC45*, a core component of the CMG complex, the replicative helicase that travels with the replisome during S phase [26,43]. A specific missense mutation (P463L) occurs at an evolutionary conserved position of the human Cdc45 protein [26]; therefore, we introduced the equivalent MGS mutations into budding yeast (*cdc45*^P542L^). We analyzed the chromosome XII size of five *cdc45*^P542L^ isolates immediately after selection for loss of the *CDC45* allele and found that all the isolates had shortened chromosome XII (S5 Fig). Although we observe a contraction of the rDNA locus in *cdc45*^P542L^ yeast cells, it is important to note that shortening of this locus in the *cdc45*^P542L^ and *orc4*^Y232C^ mutants analyzed may occur through different mechanisms. However, since both Cdc45p and Orc4p are required for DNA replication, we sought to determine if defects during DNA replication may be responsible for this unusual phenotype.

Because each rDNA repeat contains a potential origin of replication (rDNA ARS) [44], we asked if compromised replication initiation at the rDNA ARS could be responsible for the delay in completion of S phase and ultimately in the reduction of rDNA copy number. Replication of the rDNA locus is a bit unusual: 1) Each repeat contains a potential origin but only a subset of them—usually those rDNA ARSs downstream of transcriptionally active repeats—serve as origins in any given S phase [45]. 2) Because of the high transcriptional activity, replication is almost entirely unidirectional—enforced by the replication fork barrier (RFB) that blocks forks from entering the 3’ end of the 35S transcription unit in a direction that opposes transcription [44]. Compared to bidirectional replication elsewhere in the genome, the unidirectional replication in the rDNA would require twice the number of initiation events for the same territory to be replicated in the same amount of time. 3) The rDNA locus completes its replication late in S phase [46]. We reasoned that if origin initiation at the rDNA locus were less efficient in *orc4*^Y232C^ cells it could explain the delay in completion of S phase and the reduction in rDNA copy number because cells that had suffered a reduction in rDNA length would complete replication more quickly and their descendants would take over the culture.

**Fig 3:**
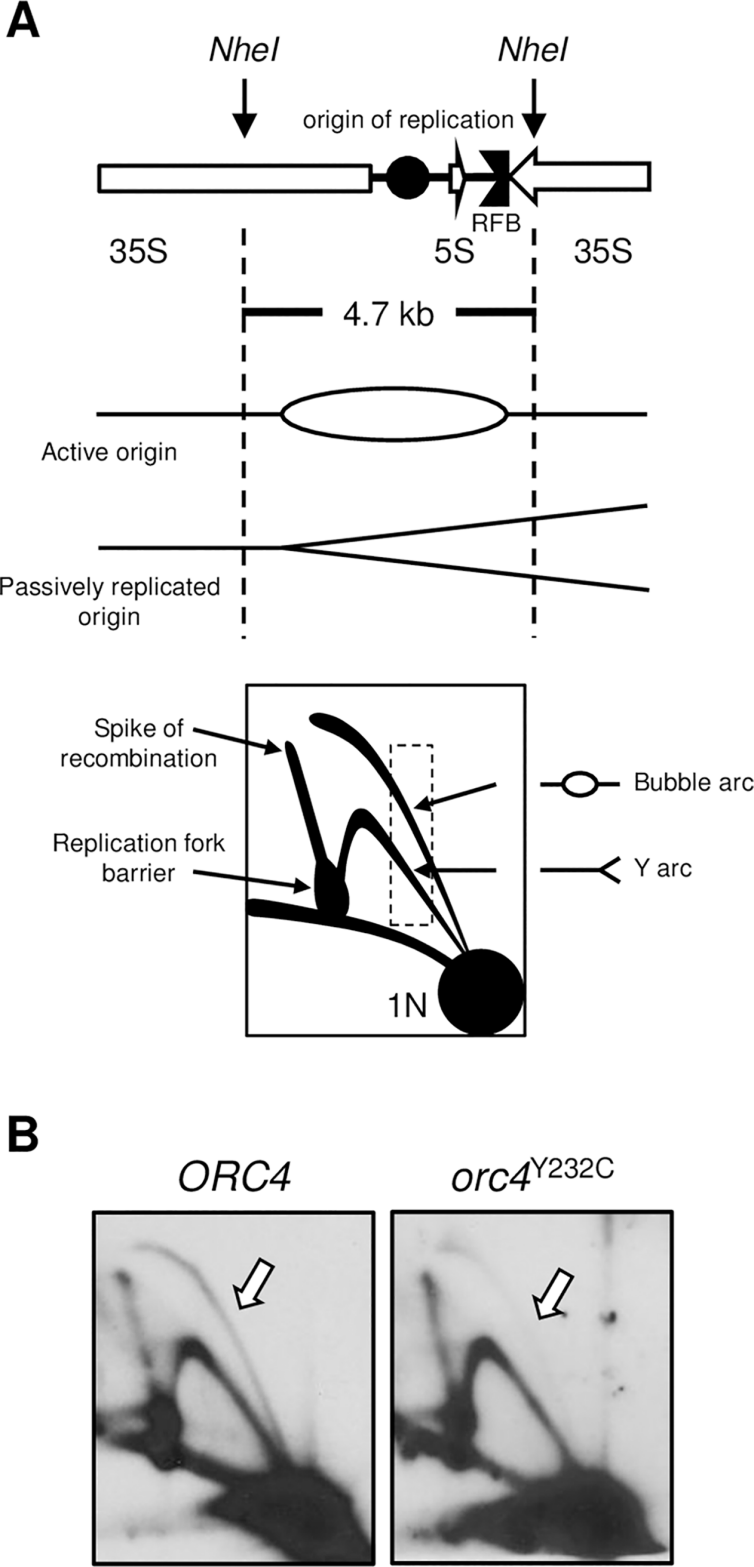
Origin initiation at the rDNA locus is reduced in *orc4*^Y232C^ cells. (A) (Top) Organization and *NheI* restriction map of a yeast rDNA repeat. Shown are the rDNA origin of replication (*rARS*) and replication fork barrier (RFB) located in the intergenic space between the 5S and 37S genes (white arrows). Replication intermediates present in the 4.7 kb *NheI* fragment are resolved using 2D-gel electrophoresis. (Bottom) Cartoon image of a 2D-gel illustrating how different replication intermediates are resolved. Relative origin efficiency is obtained by comparing intensities of replication intermediates with similar mass (dashed-line box). (B) Origin activity is reduced at the rDNA locus in *orc4*^Y232C^ cells. The 4.7 kb *NheI* fragment was excised from rDNA repeats from logarithmically growing cells and examined by 2D-gel electrophoresis. The Southern blot was probed for sequences specific to *rARS*. Bubble intermediates (white arrow) are reduced in *orc4*^Y232C^ cells compared to *ORC4* cells.

To test the efficiency of rDNA origin firing, we carried out 2D gel electrophoresis of genomic DNA from cells in logarithmic growth [47]. By focusing on the *NheI* fragment that contains the rDNA ARS at its center we can detect the fraction of rDNA repeats that gives rise to bubble intermediates (active origin) relative to the fraction of repeats in which the origin region is passively replicated by a fork moving through (inactive origin) (Fig 3A). The wild type cells showed the expected frequency of repeats with an active origin (roughly 1 in 5 repeats has an active origin; Fig 3B). In contrast, *orc4*^Y232C^ cells had a greatly reduced “bubble arc” relative to the intensities of the “Y arc”, the RFB pause site and the non-replicating “1N” spot. After adjusting for copy number differences in rDNA repeats between the two strains we conclude that origin activation within the rDNA array is a very rare event in the *orc4*^Y232C^ mutant cells.

What might be the cause for the reduced origin activity of rDNA origins in the mutant cells? One possibility is that the mutant allele results in a less stable protein, therefore limiting the amount of Orc4^Y232C^ protein available to form Pre-RCs on rDNA origins. Such is indeed the case in *orc2-1* mutant cells, where protein destabilization leads to reduced pre-RC formation; simply increasing the dosage of the mutant protein by providing an additional copy of the mutant gene on a plasmid alleviates the mutant phenotype [48]. Accordingly, we asked whether an additional copy of the *orc4*^Y232C^ allele on a centromere plasmid would provide any level of rescue of the rDNA locus contraction. An *orc4*^Y232C^ clone with ~30 rDNA repeats was transformed with plasmids containing either the *ORC4* or *orc4*^Y232C^ allele and chromosome XII size was measured after ~30 generations of growth. The plasmid copy of *ORC4* resulted in a significant increase in the rDNA locus while the same plasmid with the *orc4*^Y232C^ allele provided no rescue (S6 Fig). These results suggest that it may not be protein levels that are contributing to reduced rDNA origin firing, but rather, the rDNA origin is less efficiently activated in cells with the mutant ORC complex.

To test whether inefficient rDNA origin firing was responsible for contraction of the rDNA locus we reasoned that a compromised rDNA ARS would exacerbate the phenotype. A naturally occurring rDNA variant, obtained from the Robert Mortimer vineyard strain RM11-1a, was introduced into the laboratory background BY4741 [49]. This variant, which has a T to C transition in a highly-conserved residue of the rDNA ACS (S7 Fig), is known to reduce but not eliminate *rARS* activity [49]. Cells with this weakened rDNA variant in an otherwise wild type background show no change in growth rate (compare Fig 1A and S8 Fig). For convenience, we shall hereafter refer to the lab (BY4741) version of the rDNA as *rDNA*^BY^ and the vineyard strain (RM11-1a) variant rDNA as *rDNA*^RM^.

**Fig 4:**
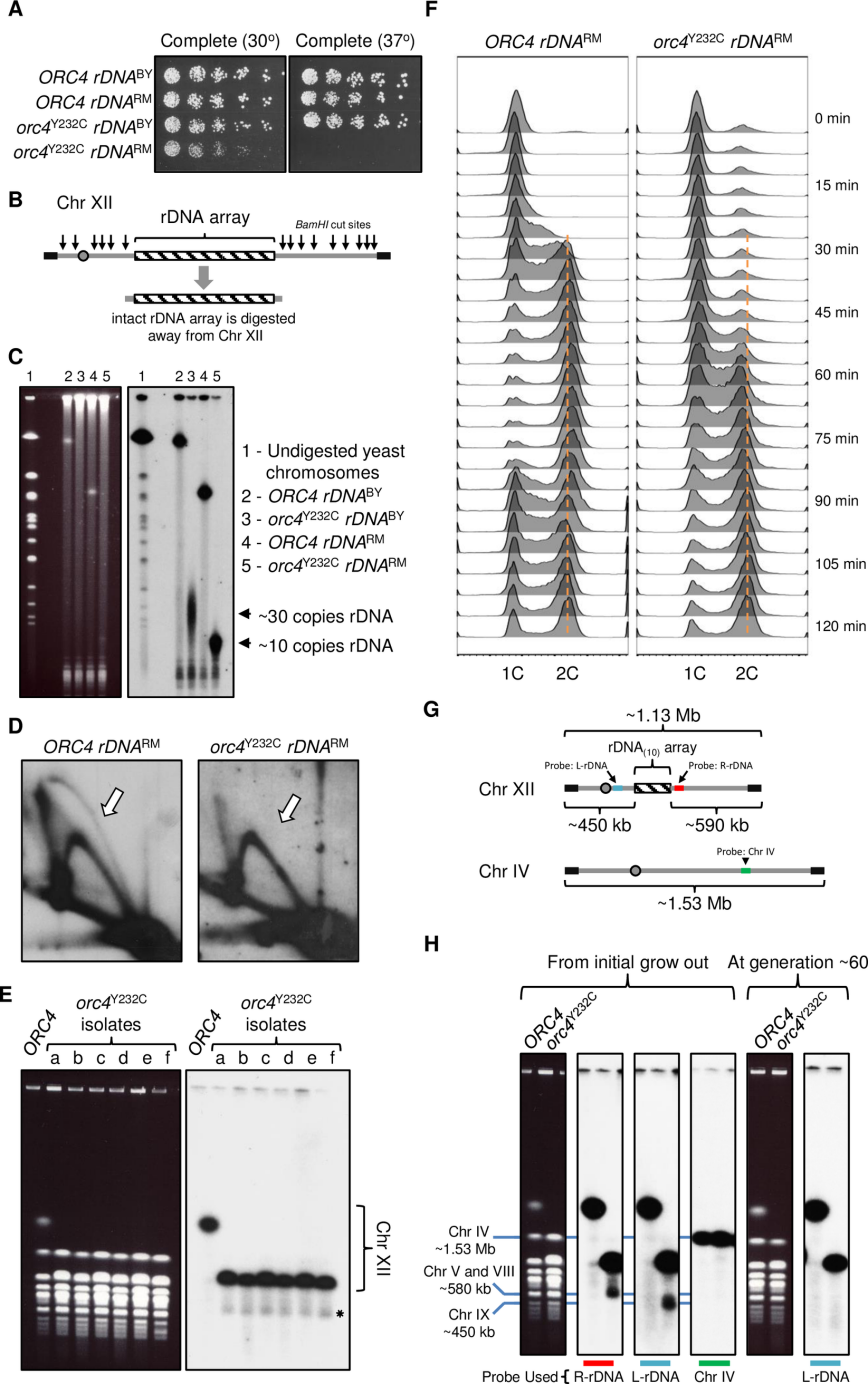
*orc4*^Y232C^ cells with a less efficient *rARS* exhibit exacerbated growth and rDNA phenotypes. (A) Slow growth and temperature sensitive phenotypes of *orc4*^Y232C^ *rDNA*^RM^ cells. Serial dilutions (1:3) of cells were plated on synthetic complete medium and incubated at either 30°or 37°C for 3 days. (B) Outline of strategy used to release intact rDNA array from Chr XII by restriction enzyme digest with *BamHI*. (C) CHEF gel electrophoresis of whole yeast chromosomes digested with *BamHI* (ethidium bromide stained image on left, Southern hybridization on right). The Southern blot was probed with a single copy sequence that is within the *BamHI* rDNA fragment and adjacent to the rDNA array. The migration of this fragment relative to undigested yeast chromosomes as size standards shows that *orc4*^Y232C^ *rDNA*^RM^ cells (lane 5) have ~10 copies of rDNA. (D) Bubble intermediates (white arrow) are not detectable at the rDNA locus in *orc4*^Y232C^ *rDNA*^RM^ cells. The 4.7 kb *NheI* fragment was excised from rDNA repeats from logarithmically growing cells and examined by 2D-gel electrophoresis. Subsequently, the Southern blot was probed for sequence specific to the *rARS*. (E) CHEF gel analysis of variation in rDNA copy number in six additional isolates (a-f) of *orc4*^Y232C^ *rDNA*^RM^. Left, ethidium bromide stained image; right, Southern blot hybridization for Chr XII as in Fig 2. Samples were prepared after ~20 generations of growth following introduction of the *orc4*^Y232C^ mutation. The rDNA copy number is estimated to be ~10 copies in all six isolates. (F) S phase progression of *ORC4 rDNA*^RM^ (left) and *orc4*^Y232C^ *rDNA*^RM^ (right) cells measured by flow cytometry. Cells were synchronously released into S phase and samples were collected at 5-minute intervals. *ORC4 rDNA*^RM^ cells enter S phase ~20 minutes after release from alpha factor, whereas *orc4*^Y232C^ *rDNA*^RM^ cells do not enter S phase until ~40 minutes after release. Additionally, by 85-minutes *ORC4 rDNA*^RM^ cells are cycling back to begin a new cell cycle, whereas *orc4*^Y232C^ *rDNA*^RM^ cells have not yet completed cell division. The orange dotted line indicates the expected DNA content for cells that have completed replication. (G) Cartoon illustration of Chr XII (containing 10 copies of rDNA (rDNA_10_)) and Chr IV. Colored boxes represent the locations of different probes used in the following panel (H). The red box corresponds to a probe specific for a single copy sequence to the right of the rDNA locus (R-rDNA) and the cyan box corresponds to a single copy sequence to the left of the rDNA (L-rDNA). The green box represents a single copy sequence located on Chr IV. (H) Chromosome breakage is specific to Chr XII in *orc4*^Y232C^ *rDNA*^RM^ cells. “From initial grow out”: Samples prepared after ~20 generations of growth following introduction of the *orc4*^Y232C^ mutation. Different images of the same CHEF gel are shown (ethidium bromide stain on the left and three images of the same Southern blot hybridized with different probes on the right). The blue lines mark various chromosomes and their approximate sizes: Chr IV is ~1.53 Mb and Chr IX is ~450 kb. Under these electrophoresis conditions, chromosomes V and VIII migrate closely together at a size of ~580 kb. The Southern blot image on the left was probed for a single copy sequence specific to the right of the rDNA locus (R-rDNA). Note the minor band (running at ~580 kb, below the major Chr XII band) in the *orc4*^Y232C^ *rDNA*^RM^ sample. The middle Southern blot image was probed for a different single copy sequence specific to Chr XII left of the rDNA locus (L-rDNA). Note the shift of the minor band in the *orc4*^Y232C^ *rDNA*^RM^ sample to a size of ~450 kb. The Southern blot image on the right was probed for a single copy sequence specific to Chr IV. “After generation ~60”: The same strain was grown for an additional ~40 generations and samples were analyzed by CHEF gel electrophoresis. Left panel, image of ethidium bromide-stained gel; right panel, Southern blot probed with L-rDNA.

Plating assays of the wild type and *orc4*^Y232C^ strains are shown in Fig 4A. While growth of the *orc4*^Y232C^ *rDNA*^BY^ strain was comparable to that of *ORC4* strains with either rDNA version at 30°C and 37°C, growth of the *orc4*^Y232C^ *rDNA*^RM^ strain was reduced at 30°C and was undetectable at 37°C. Furthermore, the slow growth phenotype of the *orc4*^Y232C^ *rDNA*^RM^ strain is recapitulated in liquid medium at 30°C (S8 Fig): relative to the wild type strain, the growth rate of *orc4*^Y232C^ *rDNA*^RM^ is reduced by 54 minutes as compared to 18 minutes for *orc4*^Y232C^ *rDNA*^BY^. We analyzed the size of the rDNA locus by measuring the *BamHI* genomic fragment from chromosome XII that contains the intact rDNA locus plus adjacent single copy sequences (Fig 4B) by CHEF gel electrophoresis and found that the rDNA locus had undergone an even greater reduction in the *orc4*^Y232C^ *rDNA*^RM^ strain compared to the *orc4*^Y232C^ *rDNA*^BY^ strain (Fig 4C)—from 30 copies to ~10 copies. An rDNA locus of this size (~91 kb) is at the upper limit of a replicon that could reasonably be expected to be replicated by a fork established at the nearest upstream origin in the flanking unique sequences [50,51]. 2D gel analysis (Fig 4D) confirmed that there is no detectable origin initiation within the rDNA locus itself. Unlike the cells containing the BY version of the rDNA ARS, all the initial *orc4*^Y232C^ isolates that contained the RM version of the rDNA ARS showed this drastic reduction in rDNA content and did not require an extended period of growth to achieve the steady-state reduction in rDNA copy number (Fig 4E). Cells released from an alpha-factor arrest into a synchronous S phase showed a similar late-S/G1 delay but an additional and exaggerated delay in entry into S phase (~40 minutes in *orc4*^Y232C^ *rDNA*^RM^ compared to ~20 minutes in *ORC4 rDNA*^RM^; Fig 4F; compare with Fig 1B).

If the prolonged G1 to S delay in *orc4*^Y232C^ *rDNA*^RM^ cells were caused by insufficient origin activation across the genome, we would anticipate the total number of early active origins to be lower in this strain. To test this hypothesis, we carried out the same ssDNA assay as before on both *ORC4 rDNA*^RM^ and *orc4*^Y232C^ *rDNA*^RM^ cells. The full set of comparisons for all the chromosomes between *ORC4 rDNA*^RM^ and *orc4*^Y232C^ *rDNA*^RM^ cells are shown in S9 Fig and quantification of the area under peaks and pairwise comparisons between the strains are shown in S10 Fig. Similar to our previous observation, we found differences in the activation of early origins between *ORC4 rDNA*^RM^ and *orc4*^Y232C^ *rDNA*^RM^ cells; however, the total number of active origins was similar between the two. Additionally, pairwise comparisons of the two *ORC4* or two *orc4*^Y232C^ strains with different rDNAs showed extremely good concordance, indicating that the rDNA ARS genotype does not affect early genome wide replication initiation dynamics. These observations suggest that the prolonged G1 to S transition observed in the cell cycle analysis was not due simply to inadequate genome-wide origin activation in *orc4*^Y232C^ *rDNA*^RM^ cells but suggest that some aspect of defective rDNA replication contributes to both the entry into and completion of S phase.

### The initiation defect in *orc4*^Y232C^ *rDNA*^RM^ leads to chromosome XII breakage

Why are *orc4*^Y232C^ *rDNA*^RM^ cells slower to complete S phase and enter cell division? Are they experiencing difficulty in replicating their chromosome XII due to reduced origin activity at the rDNA locus, and if so, what might the consequences be? In the process of measuring rDNA copy number in the different isolates of *orc4*^Y232C^ *rDNA*^RM^ we noticed an additional minor band (Fig 4E; indicated by an asterisk) that was present only in the *orc4*^Y232C^ *rDNA*^RM^ samples and that migrated faster than the predominant chromosome XII band. We ruled out the possibility that the presence of the additional band was due to cross hybridization of the probe we were using because we did not observe its presence in the lane loaded with DNA from *ORC4 rDNA*^RM^ cells. Therefore, we hypothesized that the additional band might be a result of breakage of chromosome XII, specifically at the rDNA locus. As previously mentioned, we estimated that the rDNA copy number of *orc4*^Y232C^ *rDNA*^RM^ cells is ~10 copies, which should result in a reduction in the overall size of chromosome XII to ~1.13 Mb (Fig 4G). If chromosome XII were breaking at the rDNA locus, the two resulting sizes should be ~450 kb (left of the rDNA locus) and ~590 kb (right of the rDNA locus) and we should be able to detect these two different products using probes specific to different locations along the chromosome (Fig 4G). When hybridizing the Southern blot with a probe specific to the right of the rDNA locus (R-rDNA), in addition to the full-length chromosome XII, we also detected a band that migrated with a similar mobility to chromosomes V and VIII (~580 kb) in the lane loaded with *orc4*^Y232C^ *rDNA*^RM^ DNA (Fig 4H). However, when probing the same Southern blot for a sequence to the left of the rDNA locus (L-rDNA), the minor band previously detected was not seen; instead, a new minor band appeared at a size of ~450 kb. Based on the locations of the probes used and the different sizes of minor bands detected, we believe that chromosome XII is breaking at the rDNA locus in a small population of *orc4*^Y232C^ *rDNA*^RM^ cells. To determine if chromosome breakage is a general feature observed at other chromosomes in *orc4*^Y232C^ *rDNA*^RM^ cells, we next probed the same Southern blot for a sequence on the second largest yeast chromosome (chromosome IV) and observed only a single band in both lanes (Fig 4H), indicating that chromosome breakage is specific to the rDNA locus in *orc4*^Y232C^ *rDNA*^RM^ cells. Lastly, to determine if the chromosome breakage observed in *orc4*^Y232C^ *rDNA*^RM^ cells was a transient or recurrent event, we assayed for chromosome breakage again after ~60 generations of growth and observed only the major band corresponding to the intact chromosome XII (Fig 4H). This result suggests that the locus specific breakage in *orc4*^Y232C^ *rDNA*^RM^ cells occurs during a very narrow window, shortly after loss of the *ORC4* allele.

### The further loss of rDNA repeats in *orc4*^Y232C^ *rDNA*^RM^ cells leads to a reduction in ribosomal components and an increased sensitivity to the ribosome inhibiting drug cycloheximide

The combination of the *orc4*^Y232C^ mutation and the *rDNA*^RM^ locus reduces the growth rate of cells further compared to that of the *orc4*^Y232C^ *rDNA*^BY^ strain. While reducing the size of the rDNA array could permit completion of genome replication, it may come at a cost—namely, in the ability to make enough ribosomes to support robust growth. Just what is the lower limit of rDNA repeat number a yeast cell can tolerate and still support a normal ribosome population? When the rDNA copy number in yeast is artificially reduced to ~40 copies there are no obvious negative effects on growth rate and the cells increase the density of Pol I RNA polymerases per rDNA repeat to produce levels of rRNA similar to cells with ~150 copies of rDNA [52]. However, only a finite number of polymerases can be loaded onto a single rDNA repeat before space becomes limited. Based on the estimates of Pol I density and polymerization rates, yeast cells with ~30 copies of rDNA fall just short of being capable of producing levels of rRNA needed to support a normal growth rate [52]. By these calculations, *orc4*^Y232C^ cells with only ~10 copies of *rDNA*^RM^ could be further compromised in their ability to make rRNA. Therefore, we next tested our hypothesis that in addition to replication defects, the slow growth and prolonged G1 phase observed in *orc4*^Y232C^ strains is due to an inability to meet the demand for ribosome production.

**Fig 5:**
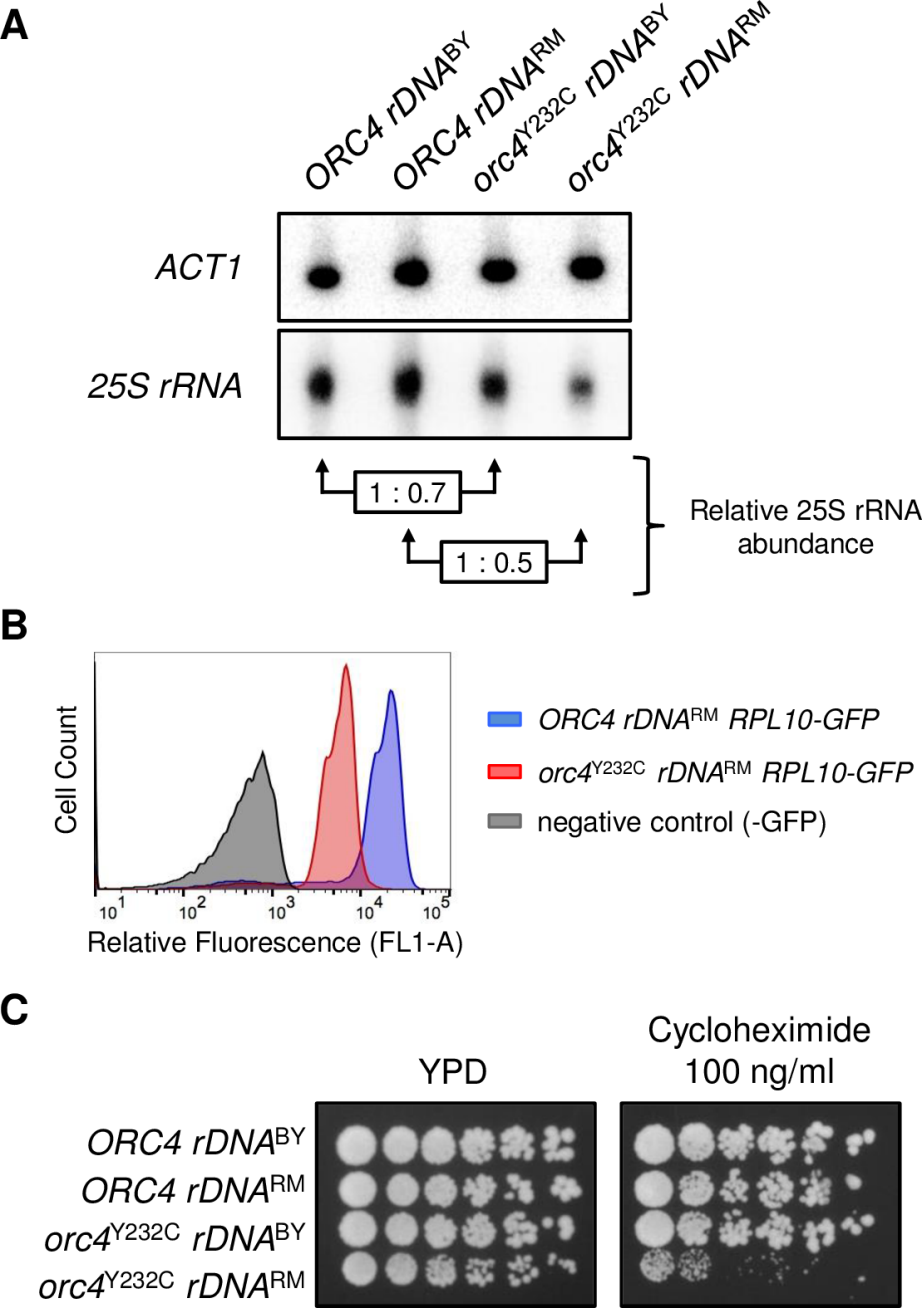
The *orc4*^Y232C^ *rDNA*^RM^ strain has reduced ribosomal components and is sensitive to the ribosome inhibiting drug cycloheximide. (A) Quantitative hybrid Southern/northern blot analysis of the 25S ribosomal RNA. Total nucleic acids (DNA+RNA) from the four strains were separated by gel electrophoresis (See S11 Fig). The top portion of the gel, containing high-molecular weight DNA, was blotted as a Southern blot and probed with *ACT1* sequence (as a single copy control). The lower portion of the gel was blotted as a northern blot and probed with *25S rRNA* sequence to measure rRNA abundance. Values representing the relative abundance of 25S rRNA in the *orc4*^Y232C^ strains were calculated by first normalizing hybridization signals of *25S rRNA* to *ACT1* in each mutant strain and then relative to its respective wild type parent. (B) Relative fluorescence of *ORC4 rDNA*^RM^ (blue) and *orc4*^Y232C^ *rDNA*^RM^ (red) cells harboring a GFP tagged version of the single-copy ribosomal protein Rpl10. Cells were grown to mid-log phase and relative fluorescence was measured by flow cytometry. (C) Sensitivity of *orc4*^Y232C^ *rDNA*^RM^ cells to cycloheximide. Serial dilutions (1:3) of cells were plated on YPD medium with or without cycloheximide (100 ng/ml) and incubated at 30°C for 3 days.

To test whether *orc4*^Y232C^ cells with a reduced rDNA copy number also have decreased levels of rRNA, we separated the total nucleic acid content from cells by gel electrophoresis (S11 Fig) and then independently transferred to hybridization membrane the two different parts of the gel containing either genomic DNA (Southern blot) or rRNA (northern blot). We found that both *orc4*^Y232C^ mutants with a reduced rDNA copy number showed a reduction in 25S rRNA levels compared to their respective wild type counterparts (Fig 5A). However, *orc4*^Y232C^ *rDNA*^RM^ cells were more severely affected, being able to make only approximately half as much 25S rRNA as wild type cells. If the drastic reduction of rRNA in *orc4*^Y232C^ *rDNA*^RM^ cells was compromising ribosome production, we proposed that ribosomal proteins would also be reduced in *orc4*^Y232C^ *rDNA*^RM^ cells. Since the per-cell fluorescence output of GFP is directly proportional to its concentration in living cells [53], we reasoned that we could determine the relative abundance of ribosomal proteins per cell by GFP labeling a ribosomal protein and then measuring the cells’ relative fluorescence using flow cytometry. Ribosomal protein levels are tightly regulated, with excess proteins being targeted for rapid destruction [54], so the abundance of ribosomal proteins is a good proxy for the abundance of ribosomes. We constructed strains of the wild type *ORC4* and *orc4*^Y232C^ mutant in the *rDNA*^BY^ and *rDNA*^RM^ backgrounds harboring a GFP-tagged version of the single-copy ribosomal protein Rpl10 (Rpl10-GFP) and measured their relative fluorescence during exponential growth by flow cytometry. The histogram of *orc4*^Y232C^ *rDNA*^RM^ cells is shifted to the left (a decrease of nearly one log) compared to wild type (Fig 5B). To determine if the shift were statistically significant, we recorded the fluorescence value for each of the 20,000 events recorded for each sample and performed a Wilcoxon Rank-Sum Test. The decrease in fluorescence we observed in *orc4*^Y232C^ *rDNA*^RM^ cells is statistically significant (p-value < 2.2e-16). We therefore conclude that there are fewer Rpl10-GFP molecules per cell in the *orc4*^Y232C^ *rDNA*^RM^ strain compared to wild type. The same decrease in fluorescence was not observed in *orc4*^Y232C^ *rDNA*^BY^ cells (S12 Fig), consistent with the relatively modest reduction of 25S rRNA measured in this strain (Fig 5A).

To investigate whether the reduction of ribosomal components in *orc4*^Y232C^ *rDNA*^RM^ cells might negatively impact their growth when translation is slightly restricted, we tested their ability to grow in the presence of a low concentration of the translation inhibiting drug cycloheximide that is tolerated by wild type *ORC4* cells. We observed that *orc4*^Y232C^ *rDNA*^RM^ cells could form colonies on the plate without cycloheximide; however, their growth was severely inhibited on the plate containing the drug (Fig 5C). This observation supports the notion that the slower growth of *orc4*^Y232C^ *rDNA*^RM^ cells and their delay in G1 is due to a lower translation capacity, rooted in their inability to make sufficient numbers of ribosomes.

## Discussion

In an attempt to uncover the link between potential chromosome replication defects and the phenotypes that characterize Meier-Gorlin syndrome we sought to identify the cellular and molecular defects conferred by the *ORC4*-MGS mutation (*orc4*^Y232C^) using yeast as a model system. We find that haploid yeast cells harboring *orc4*^Y232C^ at the endogenous chromosomal locus grow slowly, with altered S phase kinetics. Cells have reduced DNA content resulting from the loss of most copies of the nuclear 2-micron plasmid and from shrinkage of the chromosomal rDNA locus from the normal ~150 repeats to 30 repeats. Introduction of a single nucleotide variant in the rDNA origin (*rDNA*^RM^) reduces its function as an origin in *ORC4* cells and results in a further shrinkage of the rDNA locus to ~10 copies in *orc4*^Y232C^ cells. Replication initiation within the rDNA locus is greatly diminished in both rDNA types; in *orc4*^Y232C^ cells with *rDNA*^RM^, replication of the rDNA locus is thus mostly dependent on a single fork initiated at one of the adjacent, upstream origins in the unique flanking sequences. We propose that this situation is responsible for the S/G2 delay and for the growth advantage enjoyed by cells that have a shortened rDNA locus. The presence of the *rDNA*^RM^ origin exacerbates all phenotypes associated with the *orc4*^Y232C^ mutant: slower growth, further reduction in rDNA origin firing, shorter rDNA locus, chromosome XII breakage specifically within the rDNA, and a more rapid selective sweep of the short rDNA variants through the cell population. We also find that the *rDNA*^RM^ strain with the *orc4*^Y232C^ mutation experiences a drastic decrease of ribosomal components, which may account for the additional ~30 minute G1 delay before cells enter S phase.

Across the yeast genome, replication initiation is also altered by the *orc4*^Y232C^ mutation: some unique chromosomal origins share with the rDNA origin a reduction or delay in their activation but others are advanced in their replication. While we have only examined the origins that fire early in S phase, it seems unlikely, given the density of unique origins and the proportion that are affected by the *orc4*^Y232C^ allele, that there would be other stretches of the genome that would require a single replication fork to travel ~90 kb—the distance required in the truncated *rDNA*^RM^ locus. Thus, even though replication dynamics are altered genome-wide in the *orc4*^Y232C^ mutant, we conclude that difficulties in rDNA replication, resulting in a loss of rDNA repeats and ultimately a ribosome deficiency, are at the heart of the phenotypes of yeast harboring this allele.

Our work demonstrates a strong selection for reducing rDNA copy number when ORC function is compromised. A link between impaired ORC function and variation in rDNA copy number has been noted in previous studies. Ide *et al*. showed that temperature sensitive mutations in two other ORC complex proteins, Orc1 and Orc2, result in the shrinkage of the rDNA locus when cells are grown at the restrictive, or semi-restrictive temperatures [30]. In addition, they demonstrated that the shortened rDNA locus was responsible for suppressing the temperature sensitivity of these mutations. Finally, they demonstrated that replication difficulties in the rDNA triggered a Rad53 checkpoint response and that rDNA reduction attenuates this checkpoint response. Based on these observations, the authors proposed that the rDNA locus plays an important role in monitoring when origin initiation across the genome is compromised. The specific molecular event that was responsible for activation of the checkpoint response was not addressed in that study, nor was it clear why rDNA shrinkage would attenuate the checkpoint response. Our work provides new insights into the observations made by Ide *et al*. and suggests an alternative interpretation of their results. One limitation of the Ide *et al*. study is that although their model assumes reduced origin firing across the genome in the *orc* mutants, origin activity was only examined for one chromosomal origin (*ARS1*) other than *rARS*. In our broader assessment of origin activity, we find that *ORC4* and *orc4*^Y232C^ cells have a very similar number of early active origins, arguing that there is unlikely to be replication defects genome-wide. Rather, it appears that the rDNA itself is the “weak link” suffering from replication gaps. Our results suggest that as in our *orc4*^Y232C^ cells, chromosome breakage at the rDNA locus is likely responsible for triggering the Rad53 response observed by Ide *et al*.; we presume that as in the *orc4*^Y232C^ strain, the *orc1* and *orc2* cells with reduced rDNA would circumvent the rDNA replication gap problem and would therefore attenuate the checkpoint signal. Finally, although Ide, *et al*. demonstrated shrinkage of the rDNA locus partially suppressed the temperature sensitive mutations in Orc1 and Orc2 they did not explore how this rDNA shrinkage might affect rRNA synthesis. We find that having fewer rDNA repeats may allow for complete replication of this locus in *orc4*^Y232C^ cells, but at a cost. Losing too many rDNA repeats, as in the case of *orc4*^Y232C^ *rDNA*^RM^ cells, limits the amount of rRNA that can be transcribed. Therefore, *orc4*^Y232C^ cells must walk a tightrope, as it were—too many copies of rDNA and they suffer chromosome XII instability, but too few copies and they are unable to meet the demand for ribosomal RNAs.

A second study expanded on the idea that the ~150 rDNA origins compete for limiting replication initiation factors with the ~300 unique origins across the yeast genome [55]. The authors of that study discovered that this competition is regulated oppositely by two histone deacetylases—Sir2 and Rpd3. When initiation in the rDNA is increased, replication at some unique genomic origins is reduced, and vice versa. In their experiments, ORC was not the limiting factor. Instead, they showed that they could increase initiation at unique origins in a *sir2Δ* strain by overexpressing three of the initiation factors known to be in limiting supply (Sld7, Sld3 and Cdc45). We initially entertained the possibility that reduction of rDNA copy number in the *orc4*^Y232C^ mutant strain might restore a more favorable replication initiation balance between the rDNA and unique origins. However, three observations make this explanation unlikely. First, we did not find an overall reduction in unique chromosomal origin firing in the *orc4*^Y232C^ mutant—the number of early origins that failed to fire was similar to the number of new origins that appeared in the early firing class. Second, if competition between rDNA origins and unique origins were causing the growth defect in *orc4*^Y232C^, reducing the efficiency of the *rDNA*^BY^ origin by replacing the locus with *rDNA*^RM^ should have improved growth of the *orc4*^Y232C^ strain—instead, growth was further restricted and the only locus to suffer from failed replication initiation was the rDNA. Third, introducing a second copy of *orc4*^Y232C^ on a centromere plasmid did not produce any rescue in the size of the rDNA locus—suggesting that the Orc4^Y232C^ protein was not in limiting supply. Together, these observations led us to hypothesize that the *orc4*^Y232C^ mutation has altered the protein’s function.

What aspect of ORC function has been altered by the *orc4*^Y232C^ mutation? One possibility is that this single amino acid substitution has changed the DNA sequence recognized by the ORC complex. Origins in budding yeast share a similar core sequence called the ACS (ARS Consensus Sequence, an approximately 17 bp AT-rich sequence necessary but not sufficient for origin function) and variation in this sequence has been shown to impact origin usage [2]. Analysis of the different groups of origins did not reveal any simple pattern(s) of polymorphism within the ACS that distinguished wild type specific origins (i.e., origins that showed reduced activity in the mutant) from mutant specific origins (i.e., origins that had ssDNA peaks in the mutant but not in wild type), although we cannot rule out the possibility that such sequence differences do exist.

A second possibility is that there is some aspect of chromatin structure or nuclear architecture that is influencing origin choice in the *orc4*^Y232C^ mutant. One class of origins whose activity was influenced by the *orc4*^Y232C^ allele was those near centromeres: 11 of the 31 wild type specific origins were located within 10 kb of a centromere (S13 Fig). To determine if this apparently skewed distribution of wild type specific origins was significantly different than would be expected to occur by chance, we performed a permutation test. We randomly labeled 31 of the of the 213 origins used to generate the scatter plots (Fig 1D and S10 Fig) as wild type specific and asked whether at least 11 of those origins were within 10 kb of a centromere. In 10,000 trials of this test, we found no occurrence of 11 or more affected origins being within 10 kb of a centromere (p < 10^−4^). Relevant to this discussion is the observation that we and others have made that indicates that centromeres promote early firing time of origins in their vicinity [56–58]. The early firing of centromere proximal origins is thought to occur as a consequence of the kinetochore protein Ctf19 recruiting the S-phase kinase, DDK, to phosphorylate components of the pre-RC for replication initiation [57]. Whether the ORC complex with the *orc4*^Y232C^ variant is deficient in this interaction is unknown.

Lastly, nucleosome occupancy or transcription factor binding around the origin may be preventing ORC containing Orc4^Y232C^ protein from binding to some origins. Moving forward, it will be important to determine if the centromere itself, chromatin state, or specific proteins that make up the kinetochore are influencing these changes.

ORC is known to play important roles in cellular processes other than DNA replication; however, its role maintaining a healthy ribosome population has yet to be characterized. Here we show that properly functioning ORC is necessary for maintaining adequate ribosomal RNA levels through its role in rDNA replication. But when the function of Orc4 is compromised, leading to a loss of rDNA repeats and the capacity to make rRNA, how might a ribosome deficiency cause slow growth in cells? The obvious answer would be that cells cannot meet the demand for protein production—but how might certain processes such as translation and protein degradation be affected? Does the translation of certain housekeeping mRNAs take priority over non-essential ones or do cells manage to deal with a ribosome deficit by speeding up the rate of translation of all mRNAs equally? Ribosome profiling experiments to identify and quantify the mRNAs that are being actively translated by ribosomes will prove helpful in addressing these questions [59]. Additionally, if cells are struggling to keep up with the protein synthesis demands, it is possible that they modulate their protein turnover rates in response. Coupling mass spectrometry based proteomics with metabolic pulse-labeling or cycloheximide treatment of cells will help shed light on the rate of protein turnover and the general state of the proteome in cells with a ribosomal RNA deficiency [60].

Is our proposed model for loss of rDNA repeats relevant to higher eukaryotes? Just as in yeast, the human rDNA is present as tandemly repeated arrays; however, there are some notable differences. In yeast the rDNA locus is located on a single chromosome with two transcription units separately producing the 5S rRNA transcript and 35S rRNA precursor, which is processed into the 18S, 5.8S and 25S rRNAs [42]. In humans, rDNA clusters are located on six different chromosomes [61]. A locus near the end of the long arm of Chromosome 1 contains 50–200 copies of a 2.2 kb repeat that produces the 5S rRNA [61]. Loci at the ends of the short arms of Chromosomes 13, 14, 15, 21, and 22 contain 43 kb repeats coding for a 47S transcript that gets processed into the 5.8S, 18S and 28S rRNAs [61]. The number of repeats on each chromosome varies and individuals show a wide distribution of copy numbers at these five loci, ranging from between 10 to more than 100 repeats per locus [62]. While most chromosomal origins in humans are thought not to be defined by primary DNA sequence, replication initiation events in the 43 kb rDNA repeats are confined to the non-transcribed spacer [63]. What might be the consequences of impaired replication at the rDNA locus in higher eukaryotes? Bloom’s syndrome (BLM) is a rare autosomal recessive disorder characterized by short stature, immunodeficiency, and predisposition for cancer [64]. Individuals with BLM have mutations in the gene *BLM*, which encodes a member of the RecQ family of DNA helicases that acts during DNA replication [64]. Cells derived from individuals with BLM exhibit a high frequency of sister chromatid exchange and genomic instability [64]. Particularly, BLM cells exhibit elevated levels of instability at the rDNA locus [65]; however, the exact cause for this instability is unknown. Deletion of the *BLM* orthologue *SGS1* in budding yeast also results in increased instability at the rDNA locus [66]. Additionally, cancers in both human and mouse have also been shown to exhibit elevated levels of instability at the rDNA locus [67,68]. Stultz *et al*. found an increase in rDNA rearrangements in the majority of tumor samples they analyzed from lung and colorectal cancers, leading them to the conclusion that the rDNA locus is a recombination hotspot in some cancers [67]. Lastly, mice deficient for MCM2 (a component of the Pre-RC) develop lymphomas and exhibit elevated levels of DSBs at the 45S rDNA repeats in their genomes [68].

What could be the consequence(s) of increased instability at the rDNA locus in cancer? Recently, Xu *et al*. analyzed rDNA copy number in both human and mouse cancer genomes and contrary to their initial prediction, they found that rDNA copy number is reduced in the cancer state [69]. One explanation the authors propose for this unexpected result is that having fewer rDNA repeats may allow for more efficient DNA replication and thus greater cell proliferation in cancer. Given the fact that the rDNA locus is highly sensitive to replication stress in higher eukaryotes and its copy number can change rapidly during the disease state, a similar problem with rDNA replication—due to reduced origin initiation—could lead to a reduction in rDNA copy number and possibly limit the amount of rRNA and thus reduce ribosome levels in higher eukaryotes.

Meier-Gorlin syndrome phenotypes include a number of skeletal defects that have similarities to other, better-understood syndromes, all associated with deficiencies in ribosome biogenesis [70]. For example, Treacher Collins syndrome (TCS) can be caused by one of three different genetic mutations that are likely to affect ribosome production [71,72]. One of the TCS mutations is in *TCOF1*, a gene that encodes a protein called Treacle that is involved in rDNA transcription through its interaction with upstream binding factor (UBF) [73]. Treacle also interacts physically with human Nop56p, a component of the rRNA modifying box C/D small nucleolor ribonucleoprotein complex [74]. The other two genes, *POLR1C* and *POLR1D*, are subunits of RNA Polymerases I and III [72]. Work with mouse models of this syndrome suggest that ribosome deficiencies are reducing the migration of neural crest cells that are essential for proper craniofacial development [75]. Whether derived from neural crest cells or mesenchymal cells, chondrocytes also have a high demand for ribosomes as they divide, enlarge and produce collagen and other bone-matrix proteins [70]. In addition to TCS, Postaxial Acrofacial Dystosis (POADS), Diamond Blackfan Anemia (DBA), Roberts Syndrome (RBS), Schwachman-Diamond Syndrome (SDS), and Cartilage-Hair Hyploplasia (CHH) are distinct ribosomopathies with a common set of skeletal malformations [70]. Their genetic mutations affect ribosome production in different ways—a mutation that reduces uracil biosynthesis (POADS) [76,77], mutations in individual ribosomal protein genes (DBA) (reviewed in[78,79]), a mutation in a gene for cohesion (*ESCO2*) that in yeast (*ECO1*) reduces 18S and 28S production (RBS) [80,81], a mutation in *SBDS* (yeast *SDO1*) that reduces maturation of the 60S ribosomal subunit (SDS) [82–84], and a mutation in RMPR, the RNA component of RNase MRP, a snoRNA involved in rRNA processing (CHH) [85,86].

A ribosomopathy that is associated with a deficiency of rDNA has not yet been identified; however, one might speculate on the phenotypes that may be manifested with such a disorder [87].

Further work will be necessary to determine whether the phenotypes in *orc4*^Y232C^ yeast are consistent with cells derived from individuals with MGS. Additionally, determining if the slow growth phenotype observed in *orc4*^Y232C^ yeast is due primarily to defects in chromosome replication or a ribosome deficiency will prove necessary in elucidating how mutations in proteins necessary for origin initiation may inadvertently affect cellular processes in addition to DNA replication.

## Materials and methods

### Yeast strains and plasmids

A complete list of yeast strains and plasmids can be found in the S1 Table. BY4741 was used as wild type (*ORC4)* for this study and all strains were derived from this background. The rDNA locus from the Robert Mortimer vineyard strain RM11 was introduced into the BY4741 background by standard backcrossing (10 times) to create *ORC4* rDNA^RM^ [49]. Subsequently, the MGS-like variant *orc4*^Y323C^ was introduced into either *ORC4 rDNA*^BY^ or *ORC4 rDNA*^RM^ by two-step gene replacement [88]. A plasmid containing *URA3* and the *orc4*^Y232C^ allele was integrated at the *ORC4* locus; correct integrants were confirmed by PCR and Southern analysis. We selected for loss of the integrated sequences through homologous recombination by selecting against the *URA3* gene on plates containing 5-FOA. To screen for clones that had lost the wild type *ORC4* allele and had kept the *orc4*^Y232C^ allele we performed PCR using an allele specific oligonucleotide as one of the PCR primers. The MGS-like allele *cdc45*^P542L^ was introduced into BY4741 using CRISPR-Cas9 following the steps described by Laughery et al [89]. For all experiments, yeast cultures were grown at 30°C in synthetic complete medium supplemented with 2% glucose unless stated otherwise.

### Structural analysis of protein models

Protein structure visualization was performed using UCSF Chimera [90].

### Flow cytometry for cell cycle analysis

Cell cycle progression was examined using flow cytometry. Early log phase cells (OD_660_ ~0.25) were arrested in G1 by the addition of alpha factor at a final concentration of 3 μM. When >90% of the population was un-budded (time equivalent to ~1.5 population doublings), cells were synchronously released into S-phase by the addition of Pronase (Calbiochem) at a final concentration of 0.3 mg/ml. After the release into S phase, cells were harvested at 5-minute intervals, mixed with sodium azide at a final concentration of 0.1% and fixed with 70% ethanol. Cells were prepared for flow cytometry as previously described [32]. Flow cytometry was performed on a BD FACSCanto II and data were analyzed using FlowJo software.

### Contour-clamped homogeneous electric field (CHEF) gel analysis

Stationary phase cells were embedded in agarose plugs and prepared using standard procedures [91]. CHEF gel analysis of whole yeast chromosomes was performed using a BioRad CHEF-DR II Pulsed Field Electrophoresis System. Whole chromosomes were resolved in 0.8% LE agarose gels with a switch time ramped from 300–900 seconds at 100 volts for 68 hours in 0.5X TBE at 14°C. *BamH*I digested chromosomal DNA fragments were resolved in 1.0% LE agarose gels with a switch time ramped from 47–175 seconds at 165 volts for 62 hours in 0.5X TBE at 14°C. Gels were stained with ethidium bromide and photographed. Southern blotting and hybridization were performed using standard procedures.

### Two-Dimensional (2D) agarose gel analysis

Mid-log phase cells were harvested and embedded in 0.5% low melt agarose (SeaPlaque) in 50 mM EDTA and prepared as previously described [92] with slight modifications (see http://fangman-brewer.genetics.washington.edu/plug.html). DNA was subsequently digested in-gel using *NheI* (NEB). 2D gel electrophoresis was used to visualize the relative abundance of replication intermediates and was performed as described by [47]. Gels were blotted and hybridized with a probe specific to the rDNA origin of replication.

### Quantitative Southern blotting

DNA was harvested from stationary phase cells via the “Smash-and-grab” DNA isolation protocol [93] and subsequently digested with *EcoRV* (NEB). Digested DNA was separated by electrophoresis in a 0.7% Agarose LE (GeneMate) gel and then blotted following standard Southern blotting protocols. Blots were then sequentially hybridized with probes specific to *ACT1* (single copy control), mtDNA, 2-micron plasmid, and then rDNA. The hybridization signal was analyzed using the BioRad Personal Molecular Imager and Quantity One software. Hybridization signals of repetitive sequences were first normalized to *ACT1* and then relative to wild type.

### Long term growth experiments

Our initial replacement of *ORC4* with the mutant *orc4*^Y232C^ allele was performed using a “pop-in/pop-out” strategy ([88]; see Yeast strains and plasmids section above). To track long-term rDNA copy number changes following replacement of wild type with mutant *orc4*^Y232C^, we picked fresh “pop-out” candidates and inoculated liquid cultures for growth to saturation (~30 generations). After confirming the loss of *ORC4* by allele specific PCR, plugs for CHEF gel analysis were made for the *orc4*^Y323C^ positive isolates (a-f; Fig 2D). From the initial overnight cultures, a 1/100 dilution was made into 5 ml of fresh medium and allowed to grow to saturation (~7 generations). Growing of cells to saturation and then diluting back into fresh medium was performed for a total of 10 times which accounted for a total of ~100 generations of growth. In each round, cells were harvested when the culture reached saturation and embedded in agarose plugs for CHEF gel analysis.

### Quantitative hybrid Southern/northern blotting

The relative abundance of 25S ribosomal RNA was measured by quantitative hybrid Southern/northern blotting. The total nucleic acid content of cells was extracted using a version of the “Smash-and-grab” DNA isolation protocol [93] with the modification that cell walls were enzymatically disrupted using Zymolyase - 20T (Amsbio) instead of glass beads. Nucleic acids were resuspended in TE pH 8 and then separated by electrophoresis in a 1.5% LE agarose gel with ethidium bromide (0.3 μg/ml). After the ribosomal RNA was separated away from genomic DNA, the gel was photographed and then cut to separate the two portions containing genomic DNA and rRNA. Subsequently, the two different portions of the gel were blotted following standard Southern (genomic DNA) and northern (rRNA) blotting protocols. The Southern blot was probed for *ACT1* as a single copy control and the northern blot was probed for *25S rRNA* sequence. Because the amount of rRNA on the blot could be in excess of the probe, hybridization of the northern blot was limited to 2 hours to ensure that the hybridization signal was proportional to the amount of target sequence. The hybridization signals were analyzed using a Bio-Rad Personal Molecular Imager and Quantity One software. The *25S rRNA* hybridization signal was first normalized to *ACT1* and then relative to wild type.

### Flow cytometry analysis for cells expressing GFP tagged Rpl10

The GFP fluorescence of living cells was measured using flow cytometry. Strains were grown overnight in synthetic complete medium at 30°C. Fresh cultures were made by diluting these overnight cultures back to a starting OD_660_ of ~0.05. When cultures reached an OD_660_ of ~0.6, cells were diluted and sonicated, then analyzed directly using a BD Accuri C6 flow cytometer. Flow cytometry data was exported and analyzed using FloJo software.

### Mapping of single stranded DNA

A detailed protocol for this assay has previously been published [94]. Cells growing in log phase (OD_660_ ~0.25) were arrested in G1 by the addition of alpha factor and then synchronously released into S phase in the presence of 200 mM HU. Samples were collected every 15 minutes after release into S phase and cells were embedded in agarose plugs and then spheroplasted. The ssDNA from either S phase or G1 control samples were then differentially labeled with either Cy5- or Cy3-dUTP by in-gel random-primed labeling using exo- Klenow polymerase (NEB) without denaturation of template. The differently labeled DNAs were then collected and co-hybridized to Agilent G4493A yeast 4x44K ChIP to chip DNA microarrays according to the manufacturer’s recommendations. The data from scanned microarrays was extracted using Agilent’s Feature Extraction software. The ssDNA microarray data are available at NCBI GEO under accession no. GSE104671.

### ssDNA peak area script description

Areas under ssDNA peaks were assessed from Loess-smoothed microarray data (coordinates spaced 500 bp apart) using a custom Python script and a reference list of origins and their locations from OriDB [95] (http://cerevisiae.oridb.org/). Since most of the genome is double-stranded and therefore not a template for ssDNA labeling, the mean genome-wide signal was used as a “threshold” value for each sample. The “ssDNA peak area” for each origin was then calculated as a sum of S/G1 value at the origin’s location and the sequentially-added S/G1 values from adjacent data points until three “below threshold" values were reached on each side of the origin. The script does not distinguish overlapping origin peaks and therefore overlapping early firing origins in close proximity were manually curated and excluded from the analysis. This Python script is available as a supplemental file (S1 File).

## Supporting information

S1 Fig Structural comparison of *H*. *sapiens* and *S*. *cerevisiae* ORC subunits. Comparison of the structural models of *H*. *sapiens* (Hs; top panel) and *S*. *cerevisiae* (Sc; middle panel) origin recognition complex subunits (Orc1, 2, 3, 4, and 5; PDB IDs 5UJM and 5UDB, respectively). The bottom panel shows a superimposition of the Hs and Sc complexes. Data are from [8] and [9].
(TIF)

S2 Fig Position of *H*. *sapiens* Orc4-Tyr174 and *S*. *cerevisiae* Orc4-Tyr232. (A).
Superimposition of the Hs and Sc Orc4 subunits highlights the high degree of structural similarity between the proteins from the two species. The right panel focuses on the Tyrosine mutated in human MGS patients (Tyr174) and the corresponding Tyrosine in yeast (Tyr232) with the side chains of these amino acids displayed in red.
(B).
Structural models of ScOrc1-6 and ScCdc6 in complex with a double stranded DNA molecule. The right panel focuses on Tyr232 of ScOrc4, with the side chain of this amino acid depicted as red spheres.
(TIF)

S3 Fig Comparison of ssDNA profiles for *ORC4* and *orc4*^Y232C^ chromosomes. Genome wide ssDNA profiles for *ORC4* (black) and *orc4*^Y232C^ (red) are shown for cells after exposure to HU for 30 min. The relative ratio of ssDNA (S/G1) is plotted against chromosome coordinates (kb). A yellow circle denotes centromere locations and the positions of verified origins of replication are marked by orange triangles. The rDNA locus and adjacent flanking sequence on Chr XII (cf. 440–490 kb) were omitted due to insufficient probe coverage on the microarray slide.
(TIF)

S4 Fig The rDNA copy number in *orc4*^Y232C^ cells stabilizes at ~30 copies. Variation in rDNA copy number was analyzed in the six isolates (a-f) of *orc4*^Y232C^ after growth for ~100 generations. Change in Chr XII size was measured by CHEF gel electrophoresis. Left, ethidium bromide stained image; right, Southern blot image following hybridization with a Chr XII-specific single-copy sequence. By ~100 generations the size of Chr XII had stabilized at ~30 copies of rDNA for most of the population in all six isolates of *orc4*^Y232C^.
(TIF)

S5 Fig CHEF gel analysis of variation in rDNA copy number of five isolates (a-e) of *cdc45*^P542L^. Left, ethidium bromide stained image; right, Southern blot hybridization for Chr XII. All five isolates had a smaller Chr XII than *CDC45* due to loss of rDNA repeats.
(TIF)

S6 Fig The *orc4*^Y232C^ rDNA copy number phenotype is not rescued by an additional copy of *orc4*^Y232C^. An isolate of *orc4*^Y232C^ with ~30 copies of rDNA (lane #2) was transformed with a centromere plasmid (*pRS415*) containing a copy of either *ORC4* or *orc4*^Y232C^. rDNA copy number was analyzed in three isolates from each transformation. An increase in rDNA copy number was observed in cells transformed with the plasmid containing *ORC4*; however, no increase in rDNA copy number was observed in cells transformed with the plasmid containing *orc4*^Y232C^.
(TIF)

S7 Fig Sequence comparison of the ACS of BY4741 and RM11-1a rDNA origin, *rARS*. Red arrow indicates the polymorphism in the ACS.
(TIF)

S8 Fig The slow growth phenotype is exacerbated in *orc4*^Y232C^ *rDNA*^RM^ cells. Growth curves of *ORC4 rDNA*^RM^ and *orc4*^Y232C^ *rDNA*^RM^ cells generated by measuring the optical density over time of mid-log cultures in synthetic complete medium at 30°C. The mutant (white circle) shows a substantial growth defect with a doubling-time 54 minutes (27%) longer than wild-type cells (black circle).
(TIF)

S9 Fig Comparison of ssDNA profiles for *ORC4 rDNA*^RM^ and *orc4*^Y232C^ *rDNA*^RM^ chromosomes. Genome wide ssDNA profiles for *ORC4 rDNA*^RM^ (black) and *orc4*^Y232C^ *rDNA*^RM^ (red) are shown for cells after exposure to HU for 30 min. The relative ratio of ssDNA (S/G1) is plotted against chromosome coordinates (kb). A yellow circle denotes centromere locations and the positions of verified origins of replication are marked by orange triangles. The rDNA locus and adjacent flanking sequence on Chr XII (cf. 440–490 kb) were omitted due to insufficient probe coverage on the microarray slide.
(TIF)

S10 Fig Pairwise comparisons of the relative area under the peak at each origin measured in ssDNA profiles. The relative areas under the peaks for the four strains (*ORC4 rDNA*^BY^, *ORC4 rDNA*^RM^, *orc4*^Y232C^ *rDNA*^BY^, and *orc4*^Y232C^ *rDNA*^RM^) were measured and pair-wise comparisons of those values at each origin between the different strains are shown in the three scatter plots.
(TIF)

S11 Fig The total nucleic acid content of cells is resolved by gel electrophoresis. An ethidium bromide stained gel image of the total nucleic acid content from exponentially growing cells separated by electrophoresis. The blue dashed line indicates where the gel was cut so that the two different parts of the gel containing either genomic DNA or rRNA could be separately treated for Southern or northern transfer to hybridization membranes. The hybridization images and quantifications are shown in Fig 5A.
(TIF)

S12 Fig Relative fluorescence of *ORC4 rDNA*^BY^ and *orc4*^Y232C^ *rDNA*^BY^ cells harboring Rpl10-GFP. Relative fluorescence of *ORC4 rDNA*^BY^ (blue) and *orc4*^Y232C^ *rDNA*^BY^ (red) cells harboring a GFP tagged version of the single-copy ribosomal protein Rpl10. Cells were grown to mid-log phase and relative fluorescence was measured by flow cytometry.
(TIF)

S13 Fig Origins proximal to centromeres are less active in *orc4*^Y232C^ cells. (A) The locations of *ORC4* (blue circles) and *orc4*^Y232C^ (red circles) specific origins are shown across the sixteen yeast chromosomes aligned by their centromeres at x = 0. Centromere locations are marked by a yellow circle. (B) The locations of only the *ORC4* specific origins are shown relative to the locations of centromeres (yellow line). (C) The locations of only the *orc4*^Y232C^ specific origins are shown relative to the locations of centromeres (yellow line).
(TIF)

S1 Table Yeast strains and plasmids used in this study. (PDF)

S1 File Python script used to calculate the area under ssDNA peaks. (PY)
