# Supplementary figures and images for: Defective replication initiation results in locus specific chromosome breakage and a ribosomal RNA deficiency in yeast
Source: PLoS Genet. 2017 Oct 16;13(10):e1007041. doi: 10.1371/journal.pgen.1007041 (PMC5658192; doi:10.1371/journal.pgen.1007041)

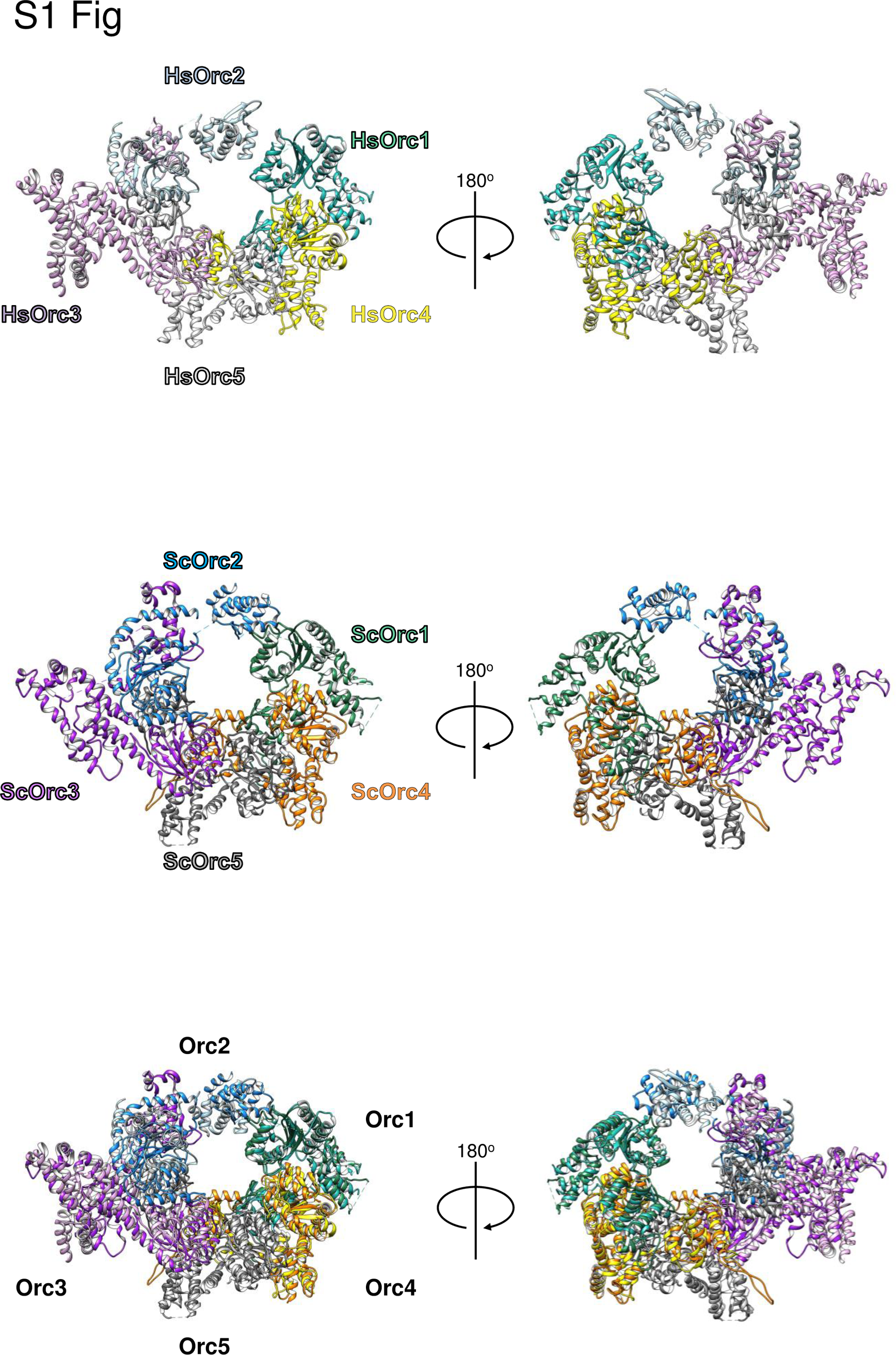

Supplement: S1 Fig — Comparison of the structural models of H. sapiens (Hs; top panel) and S. cerevisiae (Sc; middle panel) origin recognition complex subunits (Orc1, 2, 3, 4, and 5; PDB IDs 5UJM and 5UDB, respectively). The bottom panel shows a superimposition of the Hs and Sc complexes. Data are from [8] and [9]. (TIF) [file pgen.1007041.s001.tif]

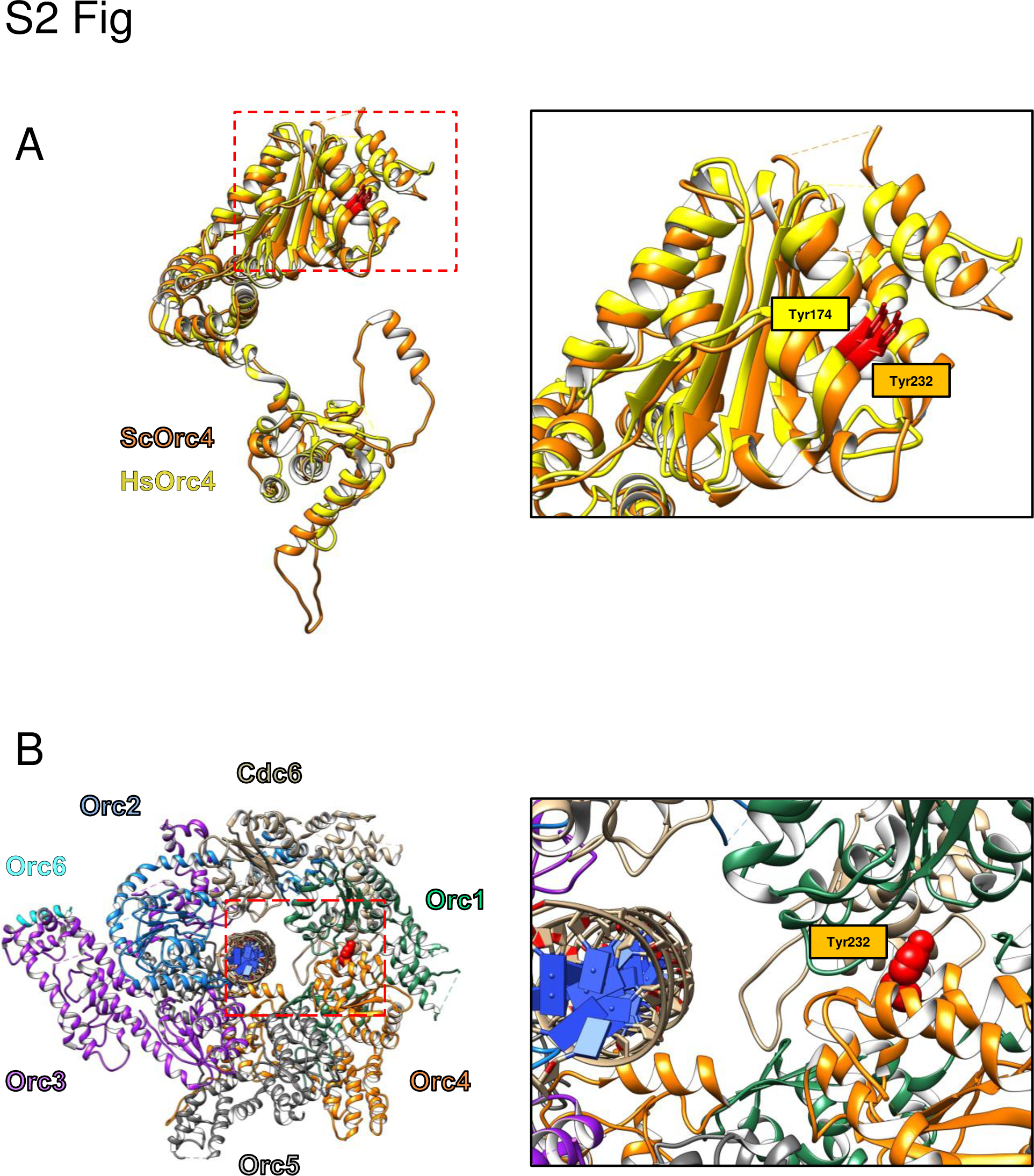

Supplement: S2 Fig — (A). Superimposition of the Hs and Sc Orc4 subunits highlights the high degree of structural similarity between the proteins from the two species. The right panel focuses on the Tyrosine mutated in human MGS patients (Tyr174) and the corresponding Tyrosine in yeast (Tyr232) with the side chains of these amino acids displayed in red. (B). Structural models of ScOrc1-6 and ScCdc6 in complex with a double stranded DNA molecule. The right panel focuses on Tyr232 of ScOrc4, with the side chain of this amino acid depicted as red spheres. (TIF) [file pgen.1007041.s002.tif]

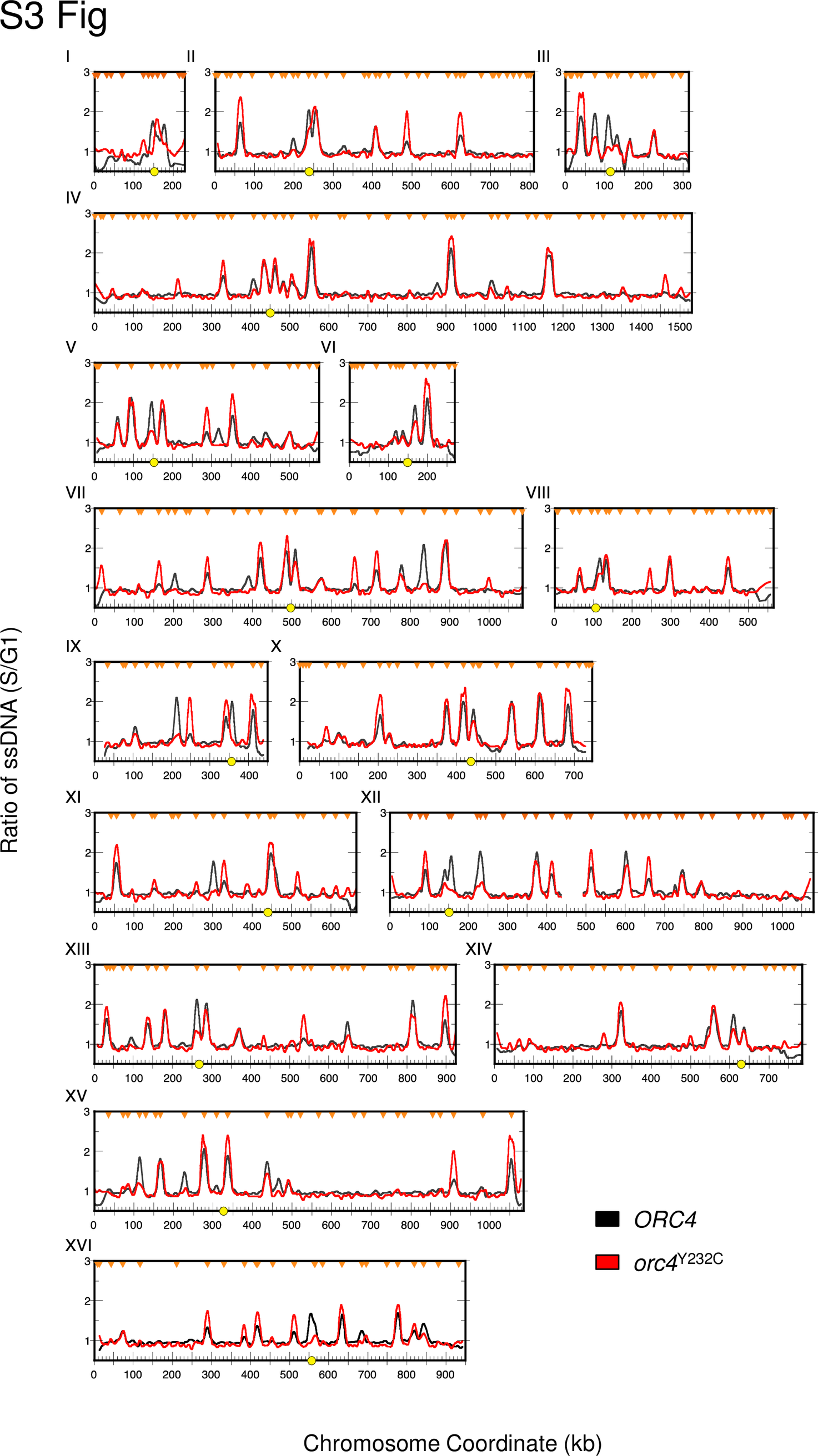

Supplement: S3 Fig — Genome wide ssDNA profiles for ORC4 (black) and orc4Y232C (red) are shown for cells after exposure to HU for 30 min. The relative ratio of ssDNA (S/G1) is plotted against chromosome coordinates (kb). A yellow circle denotes centromere locations and the positions of verified origins of replication are marked by orange triangles. The rDNA locus and adjacent flanking sequence on Chr XII (cf. 440–490 kb) were omitted due to insufficient probe coverage on the microarray slide. (TIF) [file pgen.1007041.s003.tif]

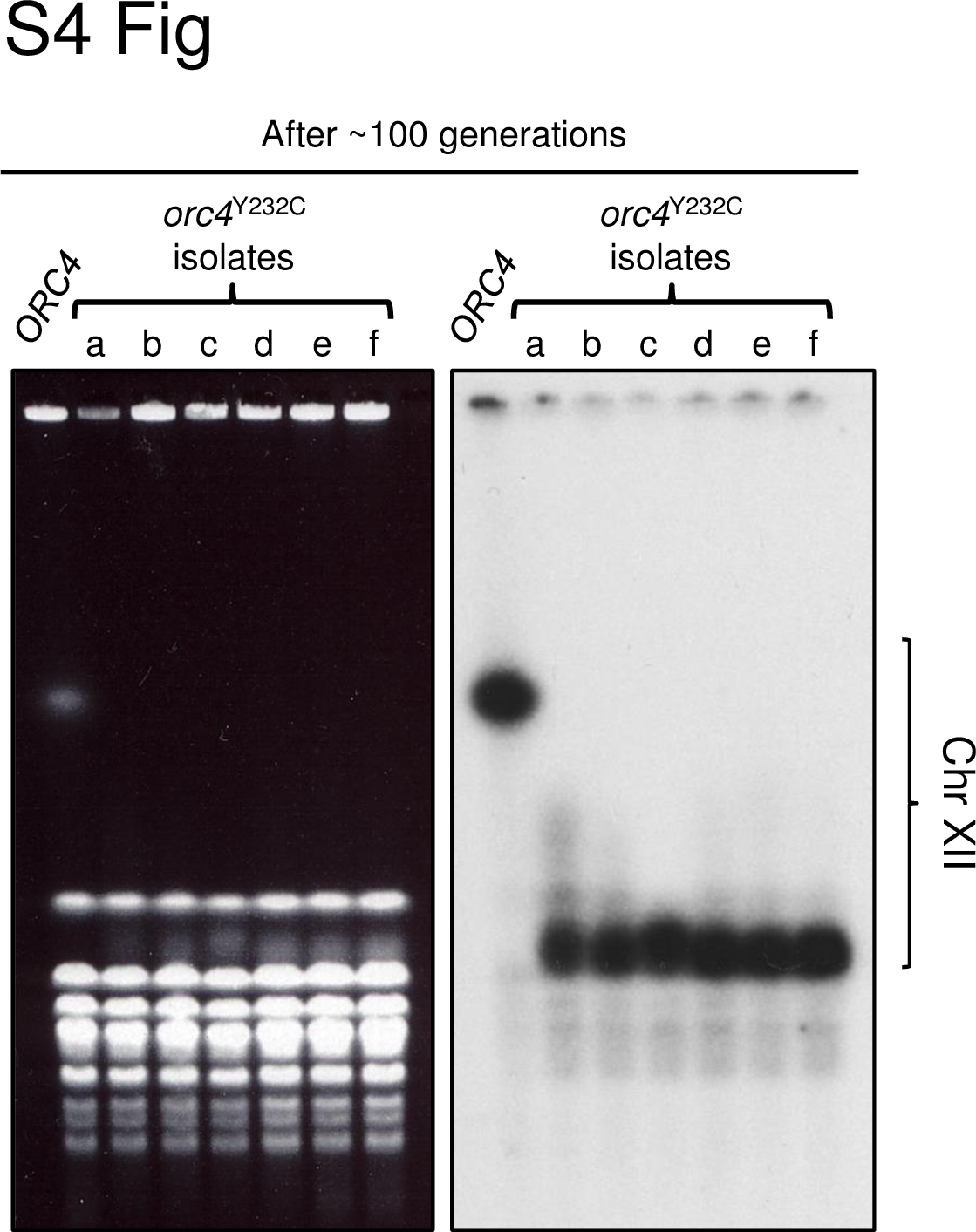

Supplement: S4 Fig — Variation in rDNA copy number was analyzed in the six isolates (a-f) of orc4Y232C after growth for ~100 generations. Change in Chr XII size was measured by CHEF gel electrophoresis. Left, ethidium bromide stained image; right, Southern blot image following hybridization with a Chr XII-specific single-copy sequence. By ~100 generations the size of Chr XII had stabilized at ~30 copies of rDNA for most of the population in all six isolates of orc4Y232C. (TIF) [file pgen.1007041.s004.tif]

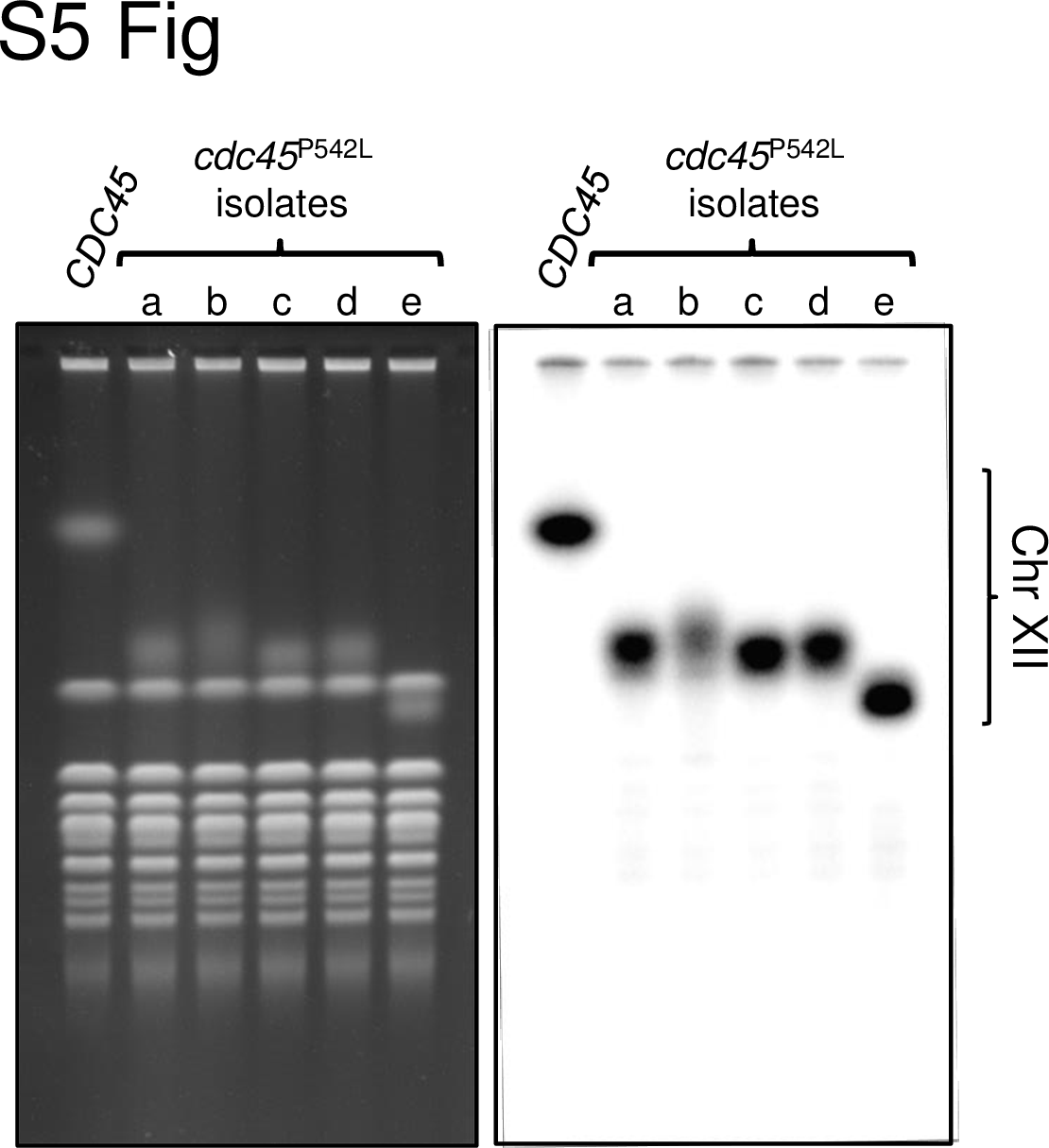

Supplement: S5 Fig — Left, ethidium bromide stained image; right, Southern blot hybridization for Chr XII. All five isolates had a smaller Chr XII than CDC45 due to loss of rDNA repeats. (TIF) [file pgen.1007041.s005.tif]

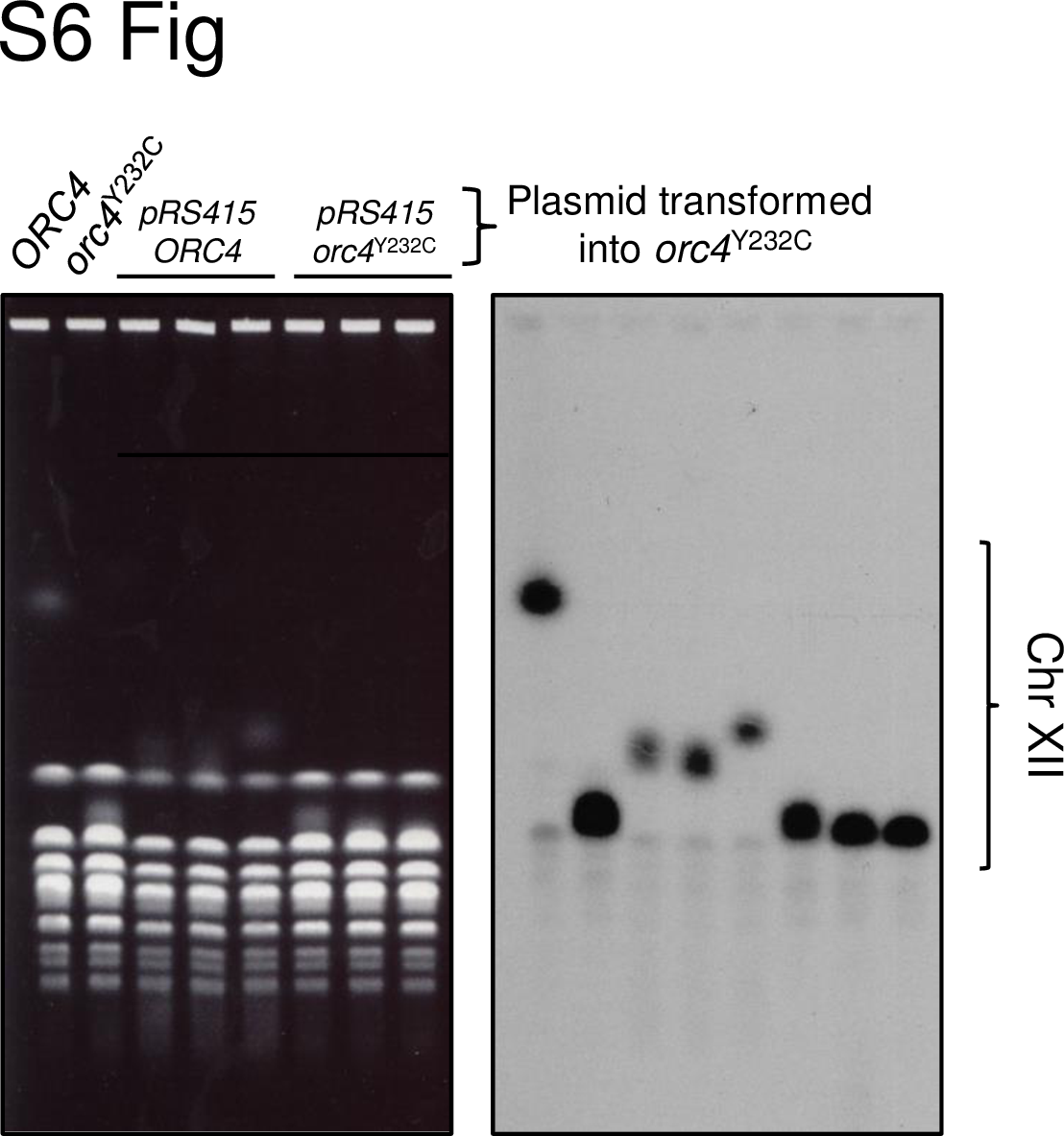

Supplement: S6 Fig — An isolate of orc4Y232C with ~30 copies of rDNA (lane #2) was transformed with a centromere plasmid (pRS415) containing a copy of either ORC4 or orc4Y232C. rDNA copy number was analyzed in three isolates from each transformation. An increase in rDNA copy number was observed in cells transformed with the plasmid containing ORC4; however, no increase in rDNA copy number was observed in cells transformed with the plasmid containing orc4Y232C. (TIF) [file pgen.1007041.s006.tif]

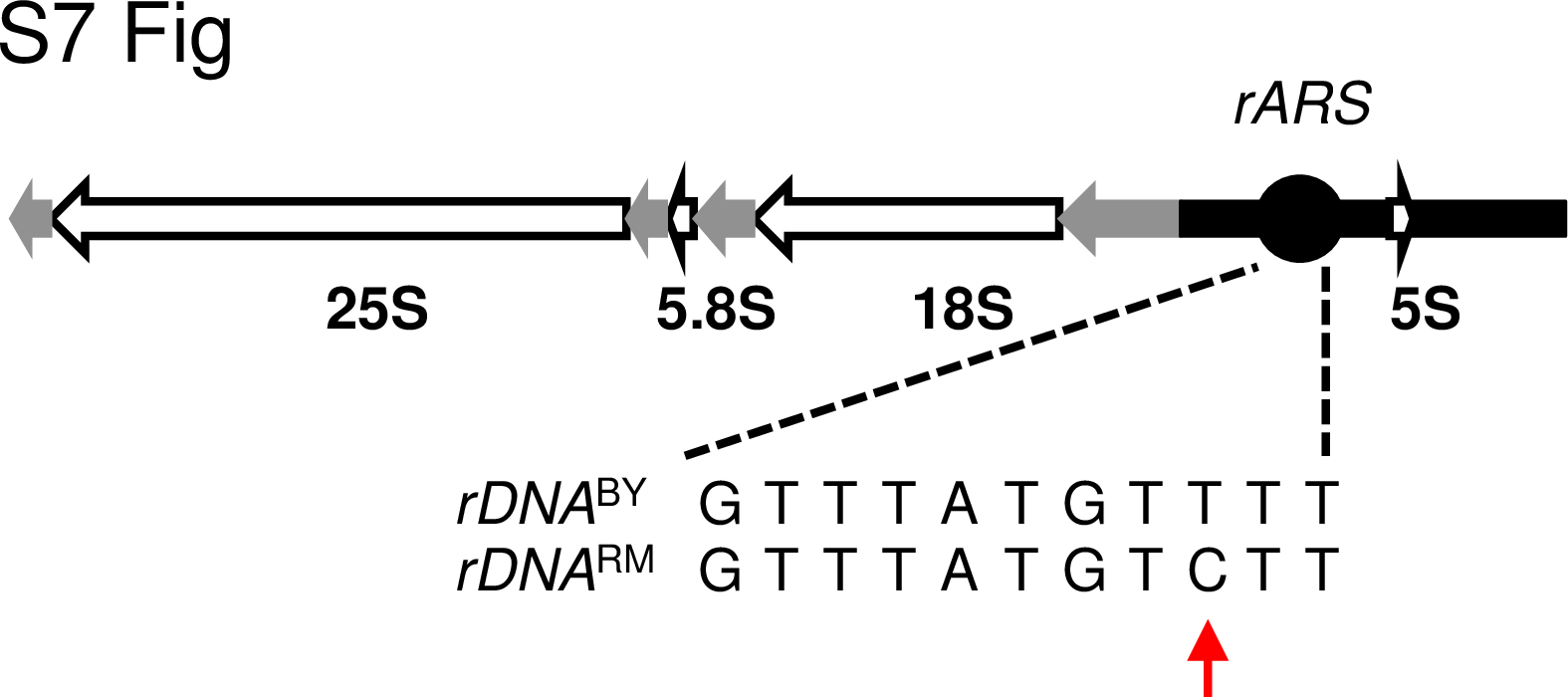

Supplement: S7 Fig — Red arrow indicates the polymorphism in the ACS. (TIF) [file pgen.1007041.s007.tif]

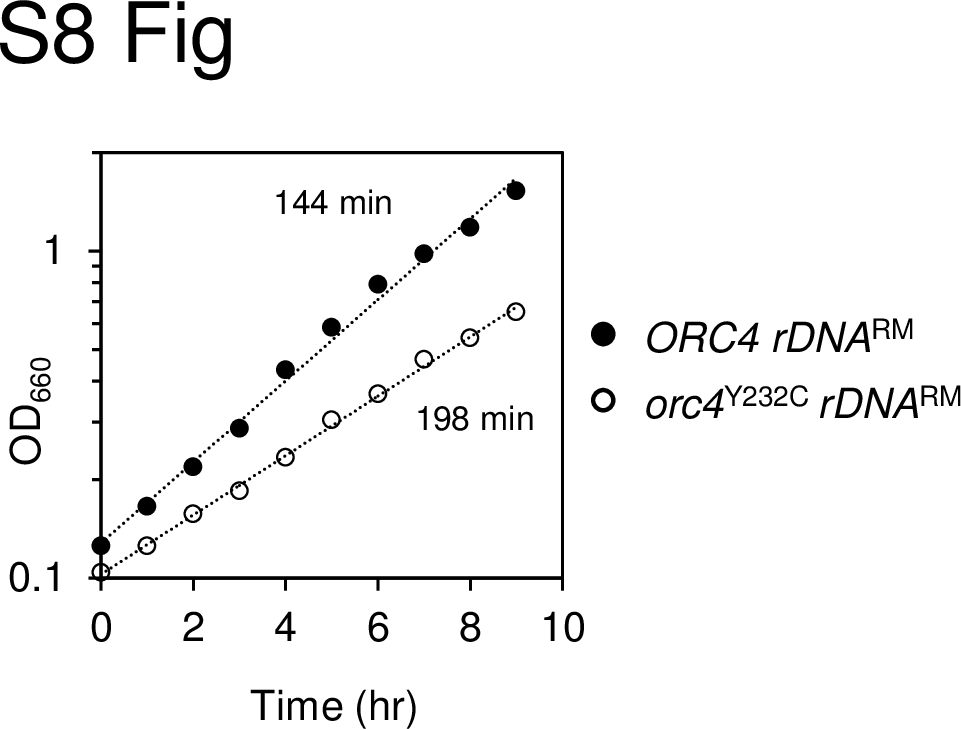

Supplement: S8 Fig — Growth curves of ORC4 rDNARM and orc4Y232C rDNARM cells generated by measuring the optical density over time of mid-log cultures in synthetic complete medium at 30°C. The mutant (white circle) shows a substantial growth defect with a doubling-time 54 minutes (27%) longer than wild-type cells (black circle). (TIF) [file pgen.1007041.s008.tif]

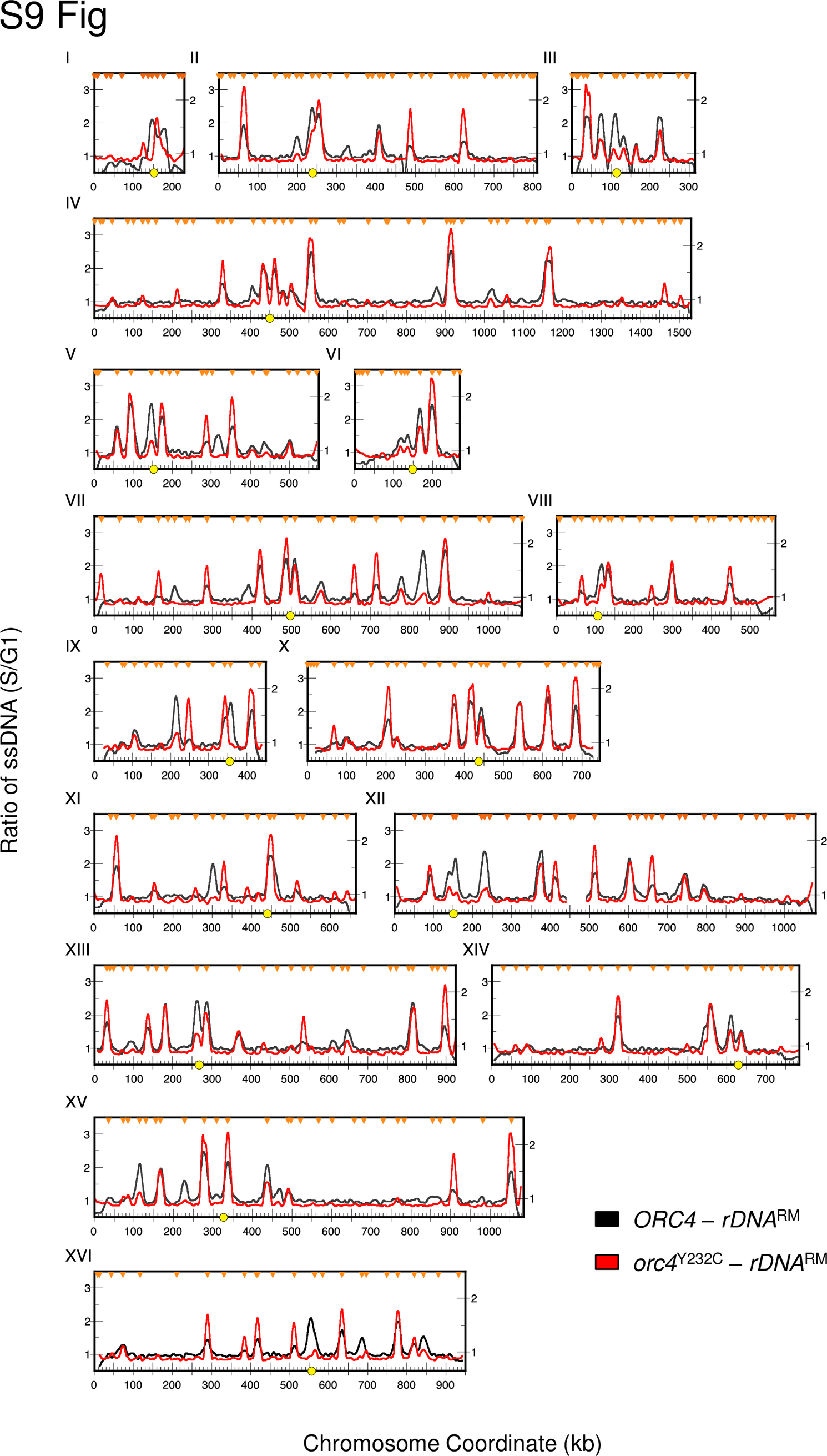

Supplement: S9 Fig — Genome wide ssDNA profiles for ORC4 rDNARM (black) and orc4Y232C rDNARM (red) are shown for cells after exposure to HU for 30 min. The relative ratio of ssDNA (S/G1) is plotted against chromosome coordinates (kb). A yellow circle denotes centromere locations and the positions of verified origins of replication are marked by orange triangles. The rDNA locus and adjacent flanking sequence on Chr XII (cf. 440–490 kb) were omitted due to insufficient probe coverage on the microarray slide. (TIF) [file pgen.1007041.s009.tif]

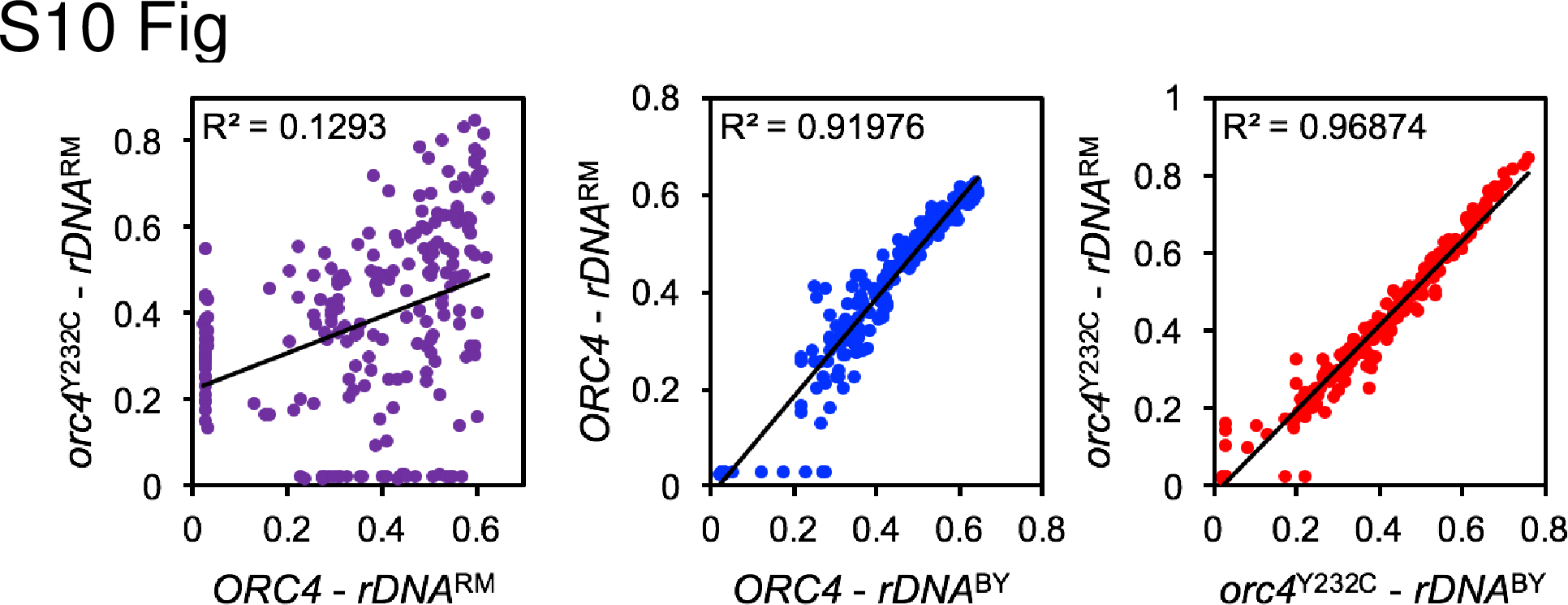

Supplement: S10 Fig — The relative areas under the peaks for the four strains (ORC4 rDNABY, ORC4 rDNARM, orc4Y232C rDNABY, and orc4Y232C rDNARM) were measured and pair-wise comparisons of those values at each origin between the different strains are shown in the three scatter plots. (TIF) [file pgen.1007041.s010.tif]

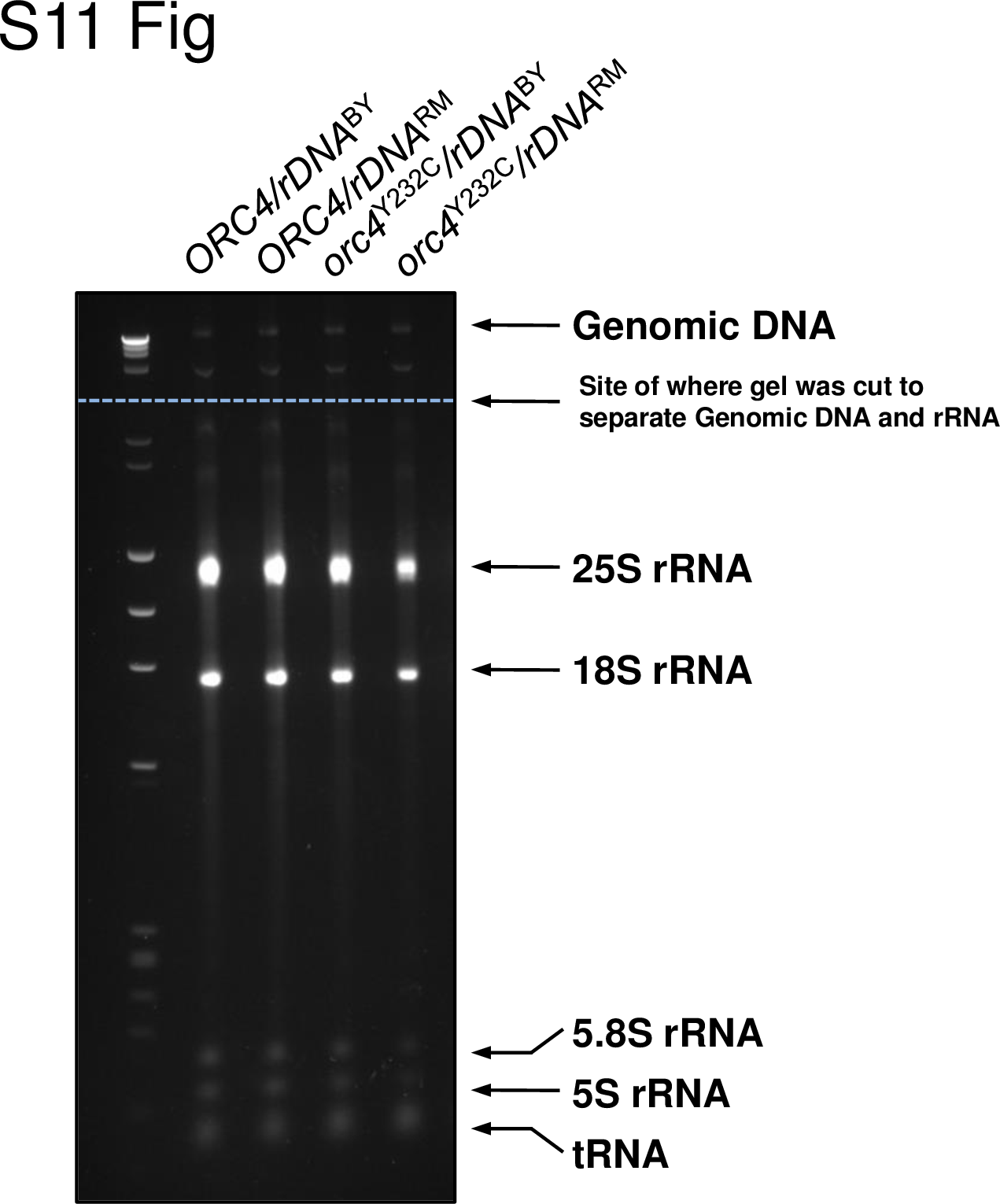

Supplement: S11 Fig — An ethidium bromide stained gel image of the total nucleic acid content from exponentially growing cells separated by electrophoresis. The blue dashed line indicates where the gel was cut so that the two different parts of the gel containing either genomic DNA or rRNA could be separately treated for Southern or northern transfer to hybridization membranes. The hybridization images and quantifications are shown in Fig 5A. (TIF) [file pgen.1007041.s011.tif]

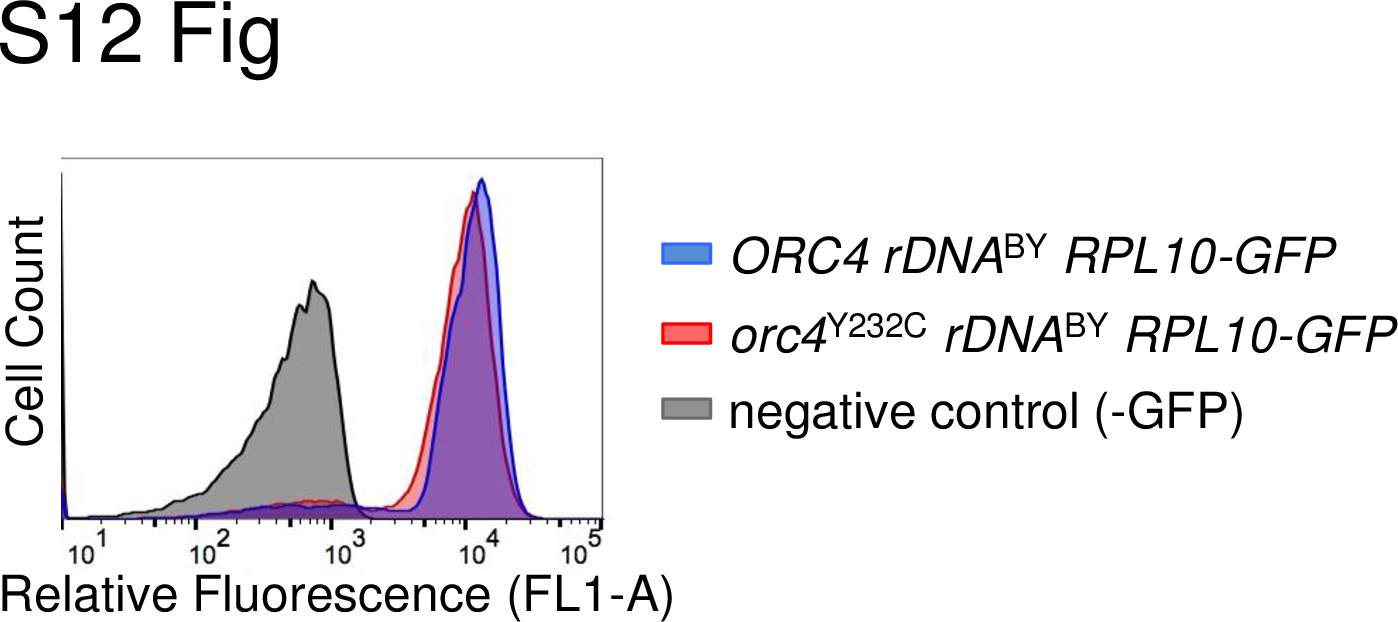

Supplement: S12 Fig — Relative fluorescence of ORC4 rDNABY (blue) and orc4Y232C rDNABY (red) cells harboring a GFP tagged version of the single-copy ribosomal protein Rpl10. Cells were grown to mid-log phase and relative fluorescence was measured by flow cytometry. (TIF) [file pgen.1007041.s012.tif]

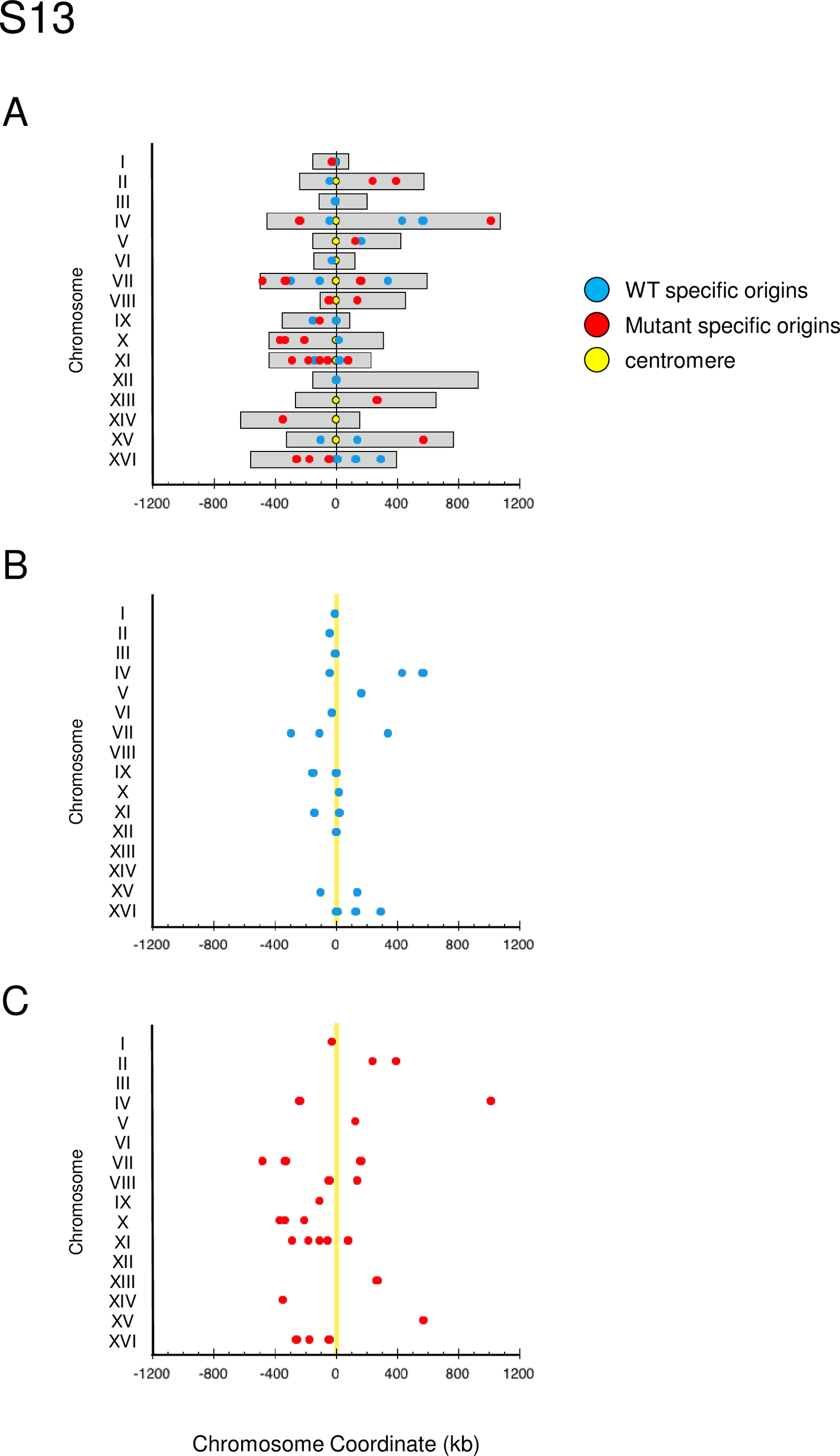

Supplement: S13 Fig — (A) The locations of ORC4 (blue circles) and orc4Y232C (red circles) specific origins are shown across the sixteen yeast chromosomes aligned by their centromeres at x = 0. Centromere locations are marked by a yellow circle. (B) The locations of only the ORC4 specific origins are shown relative to the locations of centromeres (yellow line). (C) The locations of only the orc4Y232C specific origins are shown relative to the locations of centromeres (yellow line). (TIF) [file pgen.1007041.s013.tif]
