# Supplementary material for: Defective replication initiation results in locus specific chromosome breakage and a ribosomal RNA deficiency in yeast
Source: PLoS Genet. 2017 Oct 16;13(10):e1007041. doi: 10.1371/journal.pgen.1007041 (PMC5658192; doi:10.1371/journal.pgen.1007041)
Supplement: S1 Table — (PDF) [file pgen.1007041.s014.pdf]

S1 Table: Yeast strains and plasmids used in this study

Name	Genotype	Source
BY4741 (*ORC4—rDNA*^{BY})	*MATa, his3Δ1, leu2Δ0, met15Δ0, ura3Δ0*	(Brachmann et al. 1998)
BY4741 w/ RM11-1a rDNA (*ORC4 —rDNA*^{RM})	*MATa, his3Δ1, leu2Δ0, lys2Δ0, ura3Δ0* The rDNA locus from RM11-1a was introduced into the BY4741 background by standard backcrossing (10 times).	(Kwan et al. 2013)
orc4^{Y232C} *—rDNA*^{BY}	*MATa, orc4*^{Y232C}, *his3Δ1, leu2Δ0, met15Δ0, ura3Δ0* The mutant *orc4*^{Y232C} allele was introduced into BY4741 using two step gene replacement (pop-in/ pop-out). See methods section.	This study
orc4^{Y232C} *—rDNA*^{RM}	*MATa, orc4*^{Y232C}, *his3Δ1, leu2Δ0, lys2Δ0, ura3Δ0* The mutant *orc4*^{Y232C} allele was introduced into BY4741 w/ RM11-1a rDNA using two step gene replacement (pop-in/pop-out). See methods section.	This study
cdc45^{P542L}	*MATa, cdc45*^{P542L}, *his3Δ1, leu2Δ0, lys2Δ0, ura3Δ0* The mutant *cdc45*^{P542L} allele was introduced into BY4741 using CRISPR-Cas9 following the steps described by Laughery et el.	This study
**Plasmids**	**Genotype**	**Source**
pRS415-*ORC4*	*Amp, LEU2, ORC4*	(Guernsey et al. 2011)
pRS415-*orc4*^{Y232C}	*Amp, LEU2, orc4*^{Y232C}	(Guernsey et al. 2011)
pRS406-*orc4*^{Y232C}	*Amp, URA3, orc4*^{Y232C}	This study
pML104	*Amp, URA3*	Laughery et al. (Addgene plasmid #67638)
